# Links between self-regulation competences and aggressive and non-aggressive antisocial behavior in middle childhood and early adolescence: a three-wave longitudinal study

**DOI:** 10.3389/fpsyg.2026.1866377

**Published:** 2026-09-23

**Authors:** Rahel Schaake, Sophie Strauß, Birgit Elsner, Rebecca Bondü

**Affiliations:** 1 Department for Developmental, Pedagogic and Family Psychology, Psychologische Hochschule Berlin, Berlin, Germany; 2 Department for Developmental Psychology, University of Potsdam, Potsdam, Germany

**Keywords:** aggression, inhibition, non-aggressive antisocial behavior, planning behavior, self-regulation

## Abstract

**Introduction:**

Self-regulation (SR) competences have consistently been associated with aggressive and non-aggressive antisocial behavior (AAB). However, their relative importance remains unclear when various SR competences are simultaneously considered and when they are related to different long-term trajectories of AAB. In addition, little is known about potential differential associations with AAB measures.

**Methods:**

This study investigated developmental trajectories of physical, relational, reactive, and proactive aggression; non-aggressive antisocial behavior; and an AAB total score in relation to multiple SR competences in 1,657 children (52% girls; aged 6–11 years; *M* = 8.36, *SD* = 0.95) from Germany across three measurement points over three years (data on ethnicity not collected).

**Results:**

Latent class growth analyses indicated four trajectories for all AAB measures, mostly a stable low, increasing, decreasing, and stable high trajectory, or four rather parallel trajectories. Multinomial logistic regressions suggested that particularly planning behavior (*OR*: 1.40–4.38) and parent-rated inhibition (*OR*: 1.24–3.76) predicted a higher probability of belonging to trajectories reflecting low AAB compared with all other trajectories. Additional analyses of variance further indicated the relevance of experimentally assessed inhibition, cognitive flexibility, and emotional reactivity ($\eta_{p}^{2}$ = 0.006–0.179).

**Discussion:**

These findings underpin that SR competences—particularly inhibition and planning—may serve as longitudinal protective factors and suggest that universal prevention programs are more effective than highly specific interventions to tackle AAB in the long run.

## Introduction

Aggressive and non-aggressive antisocial behavior (AAB) is frequent, with detrimental outcomes across the life-span. Pertinent theory suggests that various self-regulation (SR) competences may protect from AAB (Crick and Dodge, 1996; Gottfredson and Hirschi, 1990; Moffitt, 1993). Variable-centered empirical research has indeed evidenced vastly consistent negative relations with *cognitive* (e.g., inhibition), *behavioral* (e.g., planning), *affective* (e.g., emotion regulation), and sometimes *physiological* (e.g., heart rate variability) SR competences (Beauchaine and Thayer, 2015; Poland et al., 2016; Sullivan et al., 2010), but also indicated some differential links (Rohlf et al., 2017, 2018). Most research has examined only one or few SR competences at a time, thereby neglecting their potential relative importance for AAB. This however would be important to inform prevention programs about the most relevant SR aspects. In addition, despite substantial evidence for various trajectories that reflect diverging long-term tendencies in AAB, potential differential links between AAB trajectories and SR competences have hardly been examined. Accordant knowledge, however, would help to derive targeted interventions for distinct AAB subtypes and their developmental trajectories in question. Middle childhood and early adolescence are important developmental periods to examine potentially differential relations: SR competences dynamically develop due to neurocognitive maturation and growing social demands (DelGiudice, 2018), external control fades in favor of internal regulation (Wesarg-Menzel et al., 2023), different trajectories in forms and functions of aggression become evident, non-aggressive antisocial behavior becomes more relevant (Côté et al., 2007; Cui et al., 2016; Moffitt, 1993), the risk for long-term maladjustment increases (Ehrenreich et al., 2016) and developmentally-orientated prevention programs can reach most individuals in educational settings. Nonetheless, little attention has been paid to these developmental phases regarding SR competences in relation to such trajectories. The present study, therefore, examined the relations between various AAB measures and their trajectories and SR competences in middle childhood and early adolescence, using a person-centered approach and data from three measurement points in a large community sample.

## Aggression and non-aggressive antisocial behavior

Aggression is the intentional cause of harm to others against their will (Baron and Richardson, 1994). It is often classified into different forms that describe how aggression is displayed. Physical aggression aims to inflict physical harm onto others (e.g., kicking); relational aggression aims to damage others' social relationships (e.g., gossiping; Crick and Grotpeter, 1995). Boys show more physical aggression than girls, relational aggression shows no substantial gender differences (Card et al., 2008). Physical aggression peaks in early childhood (Côté et al., 2007), relational aggression increases throughout childhood as social and language skills increase (Casas and Bower, 2018).

Aggressive behavior is also classified by its underlying motives, known as functions of aggression (Crick and Dodge, 1996). Proactive aggression describes “cold-blooded” coercive attempts to achieve personal goals (Vitaro et al., 2006). It is linked to callous-unemotional traits and carelessness (Frick et al., 2003), but also high peer status (Price and Dodge, 1989). Reactive aggression describes “hot-blooded”, impulsive responses to perceived threat or provocation. It is linked to impaired social skills, peer victimization, and hostile attributions (Hubbard et al., 2010). Both functions of aggression are positively correlated and peak in early adolescence (Fite et al., 2008); gender differences seem negligible (Connor et al., 2003).

Antisocial behavior (AB) includes two correlated, yet distinct dimensions of aggressive and other non-aggressive, norm- or rule-breaking behavior (NA-AB), such as lying, cheating, vandalism, or running away (Burt, 2012). AB in general peaks in adolescence and is higher in boys than in girls (Burt et al., 2016). Aggressive and NA-AB differ by strong heredity, earlier onset, and high stability of, and pronounced gender differences in aggressive behavior as opposed to stronger social influences on, later onset and moderate stability of, and smaller gender differences in NA-AB (Burt, 2012; Carroll et al., 2023; Huesmann et al., 2009; Maughan et al., 2000; Sijtsema and Lindenberg, 2018). In addition, the dual taxonomy model and empirical findings suggest that individuals on the life-course persistent pathway tend to show both, rule- and norm-breaking as well as (physically) aggressive behavior, whereas individuals on the adolescent-limited pathway tend to show less severe, non-aggressive rule-breaking behavior (Burt et al., 2016; Carroll et al., 2023; Moffitt, 1993). We, therefore, differentiated between aggressive and NA-AB in our study. Aggressive and non-aggressive antisocial behavior show conceptual and empirical overlaps, but also well-established differences, for example regarding their mean level development, gender differences, and correlates. Thus, separately considering forms and functions of aggression as well as non-aggressive AB seems reasonable when investigating trajectories of this behavior and extends previous research. However, due to high correlations between measures and similarities in trajectory-patterns (see below for details), considering a combined total AAB score may highlight the value of inspecting separate AAB measures.

## Developmental trajectories of aggression and non-aggressive antisocial behavior

Early expressions of AAB in combination with specific competence deficits may mark potential long-term AAB development and provide starting points for tailored prevention. Empirical research has consistently identified distinct trajectories for different aspects of AAB. Studies in children and adolescents have mostly identified 3 or 4 trajectories for physical aggression, consistently including stable high and stable low trajectories as well as different combinations of moderate, increasing, or decreasing trajectories. For relational aggression, studies mostly found 2 or 3 trajectories, often including a stable low trajectory, as well as different combinations of moderate, increasing, or stable high trajectories (Cleverley et al., 2012; Côté et al., 2007; Ehrenreich et al., 2016; Kokko et al., 2006). Regarding reactive and proactive aggression, less extensive research often identified 3 or 4 trajectories, respectively, mostly including stable high and stable low as well as different combinations of moderate, increasing, or decreasing trajectories (Cui et al., 2016; Evans et al., 2021; Girard et al., 2019). Importantly, membership in trajectories that reflected stable high aggression during childhood predicted later school dropout, physical violence, delinquency, academic failure, and depression (Cleverley et al., 2012; Ehrenreich et al., 2016; Evans et al., 2021; Kokko et al., 2006). Being male, low socio-economic status (SES), young mothers, hostile parenting, high risk-taking and sensation-seeking, as well as low moral reasoning predicted membership in these trajectories of high aggression (Côté et al., 2007; Cui et al., 2016; Girard et al., 2019). However, trajectories of aggressive behavior have rarely been related to SR competences, despite their potential protective effect for this behavior and utility for intervention.

Concerning aggressive and NA-AB and related constructs, such as conduct problems and externalizing behavior, Moffitt (1993) suggested the two renown life course-persistent (stable high) and adolescence-limited trajectories; empirical research revealed additional adolescent-onset or increasing, childhood-limited or decreasing, stable moderate, and/or stable low trajectories (Bevilacqua et al., 2018; Fernandez Castelao and Kröner-Herwig, 2014; Odgers et al., 2008; Perry et al., 2018; Roisman et al., 2010). Members of trajectories reflecting high AB tended to experience more mental, physical, and economic problems than members of trajectories reflecting lower AB. They were more likely male and to show social, familial, and neurological risk factors as well as lower SR competences, such as heart rate variability, emotion regulation, attentional control, and inhibitory control than children on low trajectories, and more likely to show less emotion regulation and inhibitory control than children on decreasing trajectories (Fernandez Castelao and Kröner-Herwig, 2014; Odgers et al., 2008; Perry et al., 2018).

Only few studies have specifically examined trajectories of NA-AB. One study identified three trajectories (low, high, declining) from late childhood into adolescence for non-aggressive conduct problems (Maughan et al., 2000). One study identified three trajectories of low, moderate, and increasing vandalism and theft as well as four trajectories of low, and moderate/ adolescence/ early increasing alcohol-drug use, respectively, between ages 10 and 15 (van Lier et al., 2009), whereas another study showed four trajectories of abstaining, average, and increasing moderately high and high non-aggressive rule-breaking between ages 6 and 14 (Givens and Reid, 2019). Family and peer adversities, unsafe neighborhoods, premature birth, adverse temperamental traits, impulsivity, and low self-control were risk factors for high NA-AB (Carroll et al., 2023; Givens and Reid, 2019; Maughan et al., 2000). Given that trajectories of NA-AB and class membership of individuals consistently differed from those of physically aggressive AB in the more recent studies, separately considering NA-AB and its associations with SR competences, therefore, may help to delineate further differences.

Regarding an AAB total score, to the best of our knowledge, no study so far has investigated trajectories of a combined score of forms and functions of aggression as well as non-aggressive antisocial behavior. Few studies examined joint trajectories of forms and functions of aggression. For example, Girard et al. (2019) identified five trajectory groups of non-aggressors, low-stable, moderate-engagers, high-desisting, and high-chronic children between ages 6 and 13. Risk factors, such as coercive parenting, low maternal IQ, maternal depression, or no breastfeding were differentially related with specific trajectories, underlining the relevance of a person-centered approach to derive targeted interventions.

In sum, research on risk factors for AAB-trajectory membership rarely considered SR competences. Consequently, potential differential relations between various SR competences and different AAB measures and their trajectories remain unknown, although this would show whether tailored or general intervention programs are more appropriate. Separately considering AAB trajectories may be particularly important regarding children on decreasing or increasing trajectories: One the one hand, specific patterns of SR competences for these groups would allow for identifying children with a need for early intervention, even if their AAB levels not or only slightly deviated from a low or average group (i.e., the increasing group). On the other hand, we might be able to anticipate adaptive outcomes even in children with initially elevated AAB levels (i.e., the decreasing group), thereby allowing for reducing concerns in parents and teachers, the sensible investment of resources in individuals with an actual need of intervention, as well as protection from stigmatization potentially resulting from recommended intervention. We, therefore, first identified distinct AAB trajectories in all single AAB measures and a total score using latent class growth analysis, and then highlighted their relations with nine different SR competences *via* multinomial logistic regressions and ANOVAs. This allowed for the identification of potential trajectory-specific patterns of SR competences for different AAB measures. This way, the present person-centered approach adds to previous variable-centered approaches.

## Self-regulation (SR)

SR is not consistently defined (Bailey and Jones, 2019). The present study refers to SR as the ability to control and modulate responses in the cognitive, affective, behavioral, and physiological domain to attain future benefits. SR comprises a range of different competences from each of these four domains. Many competences overlap theoretically and empirically; others are fairly independent (Bailey and Jones, 2019). We selected competences that can be considered as central to the concept of SR in this age range (Gagne et al., 2021), represent each of the four domains, and were linked to AAB by previous research: Cognitive SR competences included *working-memory updating* (updating) that ensures the representation of relevant information in pursuit of a goal; *inhibition* that allows for repressing primary impulses that would override long-term goals; and *flexibility*, the ability to switch tasks, attentional focus, and strategies to reach desired goals (Röthlisberger et al., 2010). Affective SR competences included *emotional reactivity*, the tendency to easily experience intense emotions, and *emotion regulation*, that is, “initiating, avoiding, inhibiting, maintaining, or modulating” emotions through various strategies (Eisenberg and Morris, 2003, p. 191). These basal competences enable more complex behavioral SR competences, such as *planning*, the ability to organize present and future tasks (Miyake et al., 2000); *affective decision-making*, the modulation of risk when making decisions under uncertainty and in the context of desires; and *delay of gratification*, the ability to refuse immediate in favor of later larger rewards (Warschburger et al., 2023; Zelazo and Carlson, 2012). Physiological SR included *heart rate variability* (HRV), the adaption of time intervals between heart beats in response to external stressors reflecting parasympathetic activity (Holzman and Bridgett, 2017, for a meta-analysis).

## Links between SR competences and aggressive and antisocial behavior

Several theories suggest that SR competences should be relevant in the etiology and development of AAB: The social-information processing (SIP) model for aggressive behavior suggests that deficits in *cognitive* SR may impair the adequate processing of social information and promote aggressive responses (Crick and Dodge, 1994, 1996). Deficits in inhibition and updating likely disrupt early stages of encoding and interpreting ambiguous social cues, thereby benefitting reactive aggression. Reduced cognitive flexibility may impair later stages of generating and selecting non-aggressive responses to alleged aggressive cues and impede adequately assessing their advantages and disadvantages, thereby benefitting proactive aggression (Lemerise and Arsenio, 2000). Extensions of the SIP model also highlighted the role of *affective* aspects, such as high emotional reactivity and inadequate emotion regulation (Lemerise and Arsenio, 2000) that particularly promote reactive aggression (Crick and Dodge, 1996; Vitaro et al., 2006). SIP also suggests, that physical aggression is associated with higher impulsivity and problems in both, perception and reaction to ambiguous social cues. Relational aggression requires advanced SR competences, such as behavioral planning, that enable complex social behavior (e.g., manipulation), but may also point to impaired cognitive flexibility when behaviors other than manipulation and exclusion are not considered (Crick and Dodge, 1994; Crick and Grotpeter, 1995; Lemerise and Arsenio, 2000). Similarly, antisocial behavior has been explained by deficits in *cognitive* and *behavioral* SR, such as inhibition, flexibility, or affective decision making that are considered key mechanisms in distinguishing between children with persistent and desisting antisocial behavior (Moffitt, 1993, 2018). Finally, a lack of *behavioral* self-control (Gottfredson and Hirschi, 1990), that allows for considering and weighting up short-term vs. long-term rewards, has been described as the key ability that prevents from antisocial behavior.

Contrasting the fine-grained theoretical assumptions, empirical findings mainly showed overlaps in the relations between different AAB measures and SR (Supplementary Table S1 for an overview). Regarding forms of aggression, most studies found negative associations of both, physical and relational aggression, with *cognitive* SR of updating, inhibition, and flexibility, *affective* SR of emotion regulation, and *behavioral* SR of planning (Granvald and Marciszko, 2016; Rohlf et al., 2018; Sullivan et al., 2010); positive associations with *affective* emotional reactivity (Ersan, 2020; Hepditch, 2018); and no associations with *behavioral* affective-decision making (O'Toole et al., 2019; Poland et al., 2016) in middle childhood and adolescence. Regarding differences, some studies found negative links between *behavioral* delay of gratification and physical, but not relational aggression, and positive links between *physiological* HRV and relational, but not physical aggression (Murray-Close et al., 2017; Scarpa et al., 2000).

Similarly, regarding functions of aggression, both reactive and proactive aggression showed no relations with *behavioral* affective decision-making (Poland et al., 2016), negative associations with *behavioral* delay of gratification (Subramani, 2020), and mixed findings with *physiological* HRV (Jankovic et al., 2021; Puhalla et al., 2020). Regarding differences, behavior regulation (a composite of inhibition, flexibility, and emotional reactivity), emotion regulation, and metacognition (a composite including updating and planning) were negatively (Rohlf et al., 2017; White et al., 2013) and emotional reactivity was positively related (Hubbard et al., 2010) to reactive aggression, but not proactive aggression.

Antisocial behavior and related conduct problems (often including both, aggressive behavior and NA-AB) showed positive links with lower *cognitive* SR of updating, inhibition, and flexibility; lower *affective* SR of higher emotional reactivity (Hawes et al., 2016) and lower emotion regulation; and lower *behavioral* SR of lower planning, higher affective (i.e., less thought-through and advantageous) decision-making, and lower delay of gratification (Krueger et al., 1996; Moffitt and Henry, 1989; Ogilvie et al., 2011; Syngelaki et al., 2009); and mixed findings with physiological HRV (Beauchaine and Thayer, 2015).

Taken together, theoretical assumptions suggest at least some differences in SR deficits between different aspects of AAB, whereas empirical research found mostly similar impairments in most SR competences (except for affective decision-making and HRV) among individuals that showed physical, relational, and reactive aggression as well as antisocial behavior. In contrast, ambiguous or null effects support the notion that SR is not necessarily impaired in individuals that show proactive aggression. However, some of the expected fine-grained differences in the links between SR competences and AAB measures as well as their trajectories may have been blurred in previous research, because it rarely directly compared a larger number of clearly differentiated aggression, NA-AB, and SR measures. In addition, previous research was limited to variable-centered approaches of average effects. Thus, potential differential relations between specific SR competences and specific AAB trajectories may have been overlooked. Finally, the few existing studies that have related single SR competences to some trajectories of AAB did indicate differential relations between trajectories and SR competences (Cui et al., 2016; Perry et al., 2018).

## The present study

Building on and extending prior work, the present study examined trajectories of different AAB measures and their potential differential links with nine pivotal cognitive, affective, behavioral, and physiological SR competences across middle childhood and early adolescence as important developmental periods. Thereby, it covered SR across all important domains of psychological development. It used data from three measurement points covering a period of 3 years in a large sample of children, and included parent-, and teacher reports as well as experimental designs. The study aimed to add to the existing research by exploring the relative importance of multiple SR competences for the AAB trajectories across and within AAB measures in an age range that is highly relevant for the development of AAB.

Mainly based on the empirical findings from variable-centered research outlined above, first, we expected negative relations between *cognitive* updating, inhibition, and flexibility; *affective* low emotional reactivity and emotion regulation, as well as *behavioral* planning and delay of gratification and physical, relational, and reactive aggression as well as NA-AB (Hypothesis 1a), and positive relations between *behavioral* affective decision-making and NA-AB (Hypothesis 1b). We did not expect substantial relations between SR competences and proactive aggression. Second, given inconsistencies in previous findings regarding the exact number of relevant trajectories per AAB measure and differences in the exact patterns of the middle categories as well as the exploratory nature of trajectory analysis, we expected *at least two* trajectories for each form and function of aggressive behavior as well as for NA-AB, that would reflect stable low and high AAB (Hypothesis 2). Third, we expected individuals on trajectories reflecting high AAB to show less *cognitive* updating, inhibition and flexibility; *affective* emotion regulation; *behavioral* planning and delay of gratification; as well as *physiological* HRV; and more *affective* emotional reactivity and *behavioral* affective decision-making than individuals on the low trajectories, respectively (Hypothesis 3). We explored SR patterns for additional trajectories per AAB measure, gender (boy/girl), SES, and age as control variables; a total AAB score; and the SR competences that were not mentioned in the hypotheses.

## Method

### Participants

Participants were recruited from 33 primary schools in the Federal State of Brandenburg, Germany, for a longitudinal study on intrapersonal risk factors. There were three waves of measurement. At T1, 1,657 children (52% girls) between 6 and 11 years of age (*M* = 8.36, *SD* = 0.95) participated. Class teachers provided information on 1,424 and primary care givers provided information on 1,340 of these children. At T2, *N* = 1,612 children (52% girls, *M* = 9.11 years, *SD* = 0.93, range 7.11–11.89) participated and information from 1,175 class teachers and 1,197 primary care givers was available. At T3, *N* = 1,504 children participated (52% girls; *M* = 11.06 years, *SD* = 0.92, range 9.12–13.75) and information from 1,113 class teachers and 1,070 primary care givers was available. There was an average delay of 9.14 months (*SD* = 1.80) between T1 and T2 and of 23.83 months (*SD* = 1.66) between T2 and T3. Of the parents, 35% reported to have a higher educational degree (i.e., university or college); 18% had completed upper secondary schooling (i.e., A-levels), 41% had completed lower secondary school, and 1% had not completed schooling.

### Measures

#### Forms and functions of aggression as well as non-aggressive antisocial behavior

Class teachers of the respective school year rated the frequency of physical (e.g., “hit, shoved, or pushed peers”) and relational (e.g., “spread rumors or gossip about peers”) forms of aggression in the past 6 months *via* three items, respectively, by an adapted version of the Child Social Behavior Scale (CSBS-T; Crick, 1996) at all three measurement points. Response options ranged from 1 (*never*) to 5 (*daily*).

Teachers were asked to rate proactive (e.g., “to be the boss”) and reactive (e.g., “because someone teased or upset him/her”) functions of aggression *via* three items, respectively, if they had reported at least one form of aggression for the child with the Instrument for Reactive and Proactive Aggression (IRPA; Polmann, 2008) at all three measurement points. Response options ranged from 1 (*never*) to 5 (*always*). We fixed reactive and proactive aggression to 1 (*never*) in children for which teachers had reported no physical or relational aggression at the given measurement point to avoid loss of data.

Parents rated their child's non-aggressive antisocial behavior *via* six items (e.g., “runs away from home”) from the delinquent behavior subscale of the German Child Behavior Checklist (CBCL/4-18; Döpfner et al., 2014) and two items (e.g., “lies or cheats often”) from the conduct-problems subscale of the German Strengths and Difficulties Questionnaire (SDQ; Goodman, 1997). Response options were 1 *not true*, 2 *somewhat or sometimes true*, and 3 *very or often true*. We did not include any aggression-related items into the antisocial-behavior measure. We computed mean values for all subscales as well as a total AAB score, that is, a manifest mean score from the z-standardized means of the five single scales.

#### Working-memory updating

We assessed updating by the Digit-Span Backward Task from the Hamburg-Wechsler-Intelligenztest für Kinder (HAWIK-IV; Petermann and Petermann, 2007). The test administrator read out a series of digits and asked participants to repeat them in backwards order. Following a test trial, up to eight two-series trials were administered, starting at two digits and increasing by one per trial. Participants had to correctly repeat the sequence in reversed order to proceed to the next trial. Testing continued until participants failed both series within a trial. Scores were computed by the total number of correct sequences and ranged between 0 and 16. Higher scores indicate better SR.

#### Inhibition

We captured inhibition *via* two measures. The fruit-Stroop task (Archibald and Kerns, 1999) comprised four 25-stimuli trials: (1) blue, red, green, yellow rectangles, (2) fruits / vegetables in typical colors (e.g., red strawberries), (3) same fruits/ vegetables in gray, and (4) fruits/vegetables in incongruent colors (e.g., yellow strawberries). Participants had to name the colors of the stimuli as quickly as possible. In the last two trials, they had to name the correct color. The total time per trial was recorded. The dependent variable, the Stroop-interference score was computed by time p.4 – [(time p.1 x time p.3)/(time p. 1 + time p.3)]. Higher Stroop-interference scores indicate lower inhibition and SR. We measured parent-rated inhibition *via* 6 items (e.g., “can stop themselves from doing things too quickly.”) from the subscale inhibitory control of the Temperament in Middle Childhood Questionnaire (TMCQ; Simonds, 2006). Response options ranged from 1 (*almost always untrue*) to 5 (*almost always true*). We computed a mean score. Higher scores indicate better SR.

#### Flexibility

We measured flexibility by an adapted version of the cognitive attention-shifting task (Röthlisberger et al., 2010). In 46 trials (300–700 ms), children alternatingly fed a single- and a multi-colored fish on a computer screen by pressing a left- or right-hand key on a QWERTZ keyboard. The sides of the kinds of fishes changed randomly in half of the trials, requesting children to remember which fish they had fed in the previous trial and to change their response pattern accordingly. Flexibility scores reflect the number of correct switch-trial responses and ranged from 0 to 22. Higher scores indicate better SR. We only included children with a response time below 200 ms and three standard deviations above the mean.

#### Planning

Class teachers rated children's planning behavior *via* eight items from the subscale planning and organizing from the Behavior Rating Inventory of Executive Function (BRIEF; e.g., “does not plan tasks for school in advance”; Drechsler and Steinhausen, 2013). Response options ranged from 1 (*never*) to 5 (*always*). We computed and recoded the mean score so that a higher score indicates better SR.

#### Affective decision-making

To measure affective decision-making, we used an adapted version of the Hungry Donkey Task (Crone and van der Molen, 2004), an age-appropriate version of the Iowa Gambling Task (Bechara et al., 1994). In the computer-based task, participants assisted a hungry donkey in collecting apples by pressing keys to open one out of four doors (A, B, C, and D). They could win a marble if they collected at least 20 apples. When opened, each door contained a different amount of gained and lost apples that added to a score shown to the participants on the display. Doors A and B represented riskier choices (bigger gains with overall bigger losses), doors C and D represented safer choices (smaller gains but overall less losses). The dependent variable was calculated *via* the difference of favorable and non-favorable choices [(C+D)-(A+B)] during the last 50 trials. Higher scores indicate lower affective decision making and better SR.

#### Delay of gratification

Delay of gratification was experimentally assessed by **a**sking children to choose whether to instantly receive a small item amount or to wait a week and receive a larger item amount based on a procedure by Wulfert et al. (2002). There were two candy and two toy items (e.g. “Would you like one bonbon now or two bonbons in a week?”). Children only saw the smaller item amount. After the experiment, they were asked whether there were any items they did not like at all. The delay score reflects the sum of delayed items and could range between 0 and 4. Higher scores indicate better SR.

#### Emotional reactivity

Emotional reactivity was indicated *via* anger reactivity from the subscale anger frustration of the TMCQ (Simonds, 2006; e.g., “my child gets angry when they have trouble with a task”) and general emotional reactivity from the subscale emotional control of the BRIEF (Drechsler and Steinhausen, 2013; e.g., “My child becomes upset too easily”). Response options ranged from 1 (*almost always untrue*) to 5 (*almost always true*), respectively. We computed both subscale means and then averaged these means to create the emotional reactivity scores. Higher scores indicate lower SR.

#### Emotion regulation

Emotion regulation was assessed *via* four items modified for parent reports from the anger-regulation subscale of the FEEL-KJ (Grob and Smolenski, 2005; e.g., “When my child gets angry they do something that they enjoy”). We selected four items that loaded on one factor in a preliminary CFA and assessed three strategies to cope with anger: distraction, perseveration, and venting. Response options ranged from 0 (*never*) to 4 (*always*). The items representing perseveration and venting were reversely coded before computing the mean score. Higher scores indicate better SR.

#### Heart rate variability

We recorded resting state HRV by Polar Watches (Model RS800CX). Children placed their wrists on the device's electrodes for 3 min. Their hands were lightly taped to the device to avoid interruptions of the measurement due to sudden movements. Children were instructed to breath normally and to move as little as possible. We used the root mean square of successive difference (rmssd) HRV measure as provided by the watches (Friedman et al., 2002). We only included cases with no measurement difficulties and rmssd <160 (range: 6.2–431.1) into the analysis (*N* = 1,462). Higher values indicate better SR.

All SR competences were assessed at first measurement. See Table 1 for Reliabilities.

**Table 1:** Ranges, internal consistencies, means, standard deviations, and gender differences of all measures.

Measures	*n*	Range	α	Total *M* (*SD*)	Girls *M* (*SD*)	Boys *M* (*SD*)	*F*	$\eta_{\text{p}}^{\text{2}}$
T1 aggression								
Physical	1,401	1–5	0.93	1.49 (0.78)	1.22 (0.52)	1.79 (0.90)	214.84^*^	0.133
Relational	1,395	1–5	0.91	1.50 (0.70)	1.47 (0.69)	1.53 (0.70)	2.67	0.002
Proactive	1,365	1–5	0.88	1.59 (0.86)	1.41 (0.72)	1.80 (0.96)	74.48^*^	0.052
Reactive	1,373	1–5	0.94	1.94 (1.11)	1.69 (1.02)	2.21 (1.15)	79.88^*^	0.055
NA AB	1,322	1–3	0.57	1.08 (0.14)	1.07 (0.13)	1.10 (0.15)	15.59^*^	0.012
AAB	1,083	−0.69–4	0.84	−0.03 (0.77)	−0.21 (0.66)	0.16 (0.82)	66.32^*^	0.058
T2 aggression								
Physical	1,146	1–5	0.94	1.48 (0.77)	1.18 (0.46)	1.79 (0.90)	206.70^*^	0.153
Relational	1,139	1–5	0.92	1.55 (0.76)	1.50 (0.73)	1.60 (0.79)	4.80	0.004
Proactive	1,106	1–5	0.87	1.63 (0.87)	1.45 (0.77)	1.81 (0.94)	48.71^*^	0.042
Reactive	1,119	1–5	0.94	2.02 (1.17)	1.70 (1.01)	2.35 (1.23)	94.33^*^	0.078
NA AB	1,178	1–3	0.54	1.07 (0.12)	1.06 (0.12)	1.07 (0.13)	2.10	0.002
AAB	800	−0.7–2.57	0.82	−0.07 (0.74)	−0.26 (0.60)	0.14 (0.83)	61.08^*^	0.071
T3 aggression								
Physical	1,099	1–5	0.93	1.44 (0.75)	1.20 (0.48)	1.67 (0.89)	121.32^*^	0.100
Relational	1,068	1–5	0.90	1.48 (0.70)	1.45 (0.66)	1.51 (0.74)	1.90	0.002
Proactive	1,063	1–5	0.88	1.52 (0.85)	1.35 (0.69)	1.69 (0.95)	45.97^*^	0.042
Reactive	1,074	1–5	0.93	1.75 (1.04)	1.56 (0.90)	1.93 (1.14)	34.11^*^	0.031
NA AB	1,052	1–3	0.51	1.07 (0.12)	1.06 (0.12)	1.08 (0.12)	7.28	0.007
AAB	693	−0.63–3.29	0.80	−0.09 (0.69)	−0.23 (0.56)	0.06 (0.77)	30.65^*^	0.042
T1 SR competences								
WMU	1,641	0–16	–	6.18 (1.47)	6.15 (1.46)	6.21 (1.48)	0.56	0.000
Stroop	1,644	7.07–89.03	–	24.95 (8.78)	24.26 (8.01)	25.70 (9.50)	11.09^*^	0.007
Parent-Rated Inhibition	1,311	1–5	0.68	3.53 (0.66)	3.62 (0.63)	3.44 (0.68)	22.73^*^	0.017
Flexibility	1,641	0–22	–	15.39 (4.78)	16.04 (4.79)	14.67 (4.67)	34.25^*^	0.020
Emotional. Reactivity	1,283	1–5	0.91	2.42 (0.66)	2.39 (0.64)	2.45 (0.67)	3.16	0.002
Emotion Regulation	1,302	1–5	0.55	2.19 (0.66)	2.23 (0.65)	2.15 (0.66)	4.94	0.004
Planning	1,371	1–5	0.93	3.71 (0.89)	3.89 (0.84)	3.51 (0.91)	65.18^*^	0.045
ADM	1,643	−32–50	–	5.49 (11.43)	4.01 (9.63)	7.10 (12.93)	30.55^*^	0.018
DoG	1,549	0–4	0.60	2.79 (1.22)	2.71 (1.21)	2.88 (1.23)	8.08	0.005
HRV	1,462	7.5–159.6	–	58.59 (27.84)	55.73 (26.34)	61.62 (29.05)	16.53^*^	0.011
Age	1,657	6.23–11.33	–	8.36 (0.95)	8.33 (0.92)	8.39 (0.97)	1.56	0.001
SES	1,334	1–6	–	5.05 (1.01)	5.04 (1.00)	5.06 (1.01)	0.08	0.000

^*^p < 0.05; NA AB, non-aggressive antisocial behavior; AAB, total AAB score; WMU, working-memory updating; Stroop, Stroop interference; ADM, affective decision making; DoG, delay of gratification; HRV, heart rate variability; SES, socio-economic status.

### Procedure

Trained project members conducted the data collection at the children's schools or homes or in the university in two separate 45-min sessions. Children received small gifts and cinema vouchers for their participation, respectively. Teachers received 5€ for the class fund for each child about whom they answered the teacher questionnaires. The ethics committee of the University of Potsdam and the Ministry of Education, Youth, and Sport of the Federal State of Brandenburg, Germany, approved of the study. Children gave verbal consent and primary caregivers gave written consent for their child's participation. All participants were guaranteed confidentiality.

### Analysis

Descriptive analyses, ANOVAs, zero-order correlations, and MANOVAs were conducted using IBM SPSS Version 29. We computed means and standard deviations for all measures and Cronbach's alpha wherever applicable. We examined differences between boys and girls for all variables *via* Bonferroni-corrected single ANOVAS (*p* = 0.00167). To test Hypothesis 1, we computed zero-order correlations with two-tailed *p*-values between the T1 SR competences and T1–T3 AAB measures. To test Hypothesis 2, using M*plus* 8.4, we performed a series of latent class growth analyses (person-centered, group-based trajectory models with within-group variation fixed to zero and residual variances held equal across classes; Jung and Wickrama, 2008) separately for all AAB measures to determine the optimal trajectory number for each measure. Model fits for each solution were evaluated based on the Bayesian Information Criterion (BIC), Akaike Information Criterion (AIC), sample-size adjusted BIC (aBIC; lower values indicate better fit); parametric Bootstrap-Likelihood-Ratio-Test (pBLRT; Nylund et al., 2007), Vuong-Lo-Mendell-Rubin-Test (VLMRT; Lo et al., 2001; significant *p*-values suggest that a model with *k* classes fits the data better than a model with *k*−1 classes); and entropy (values closer to 1 indicate better class separation). We also considered the parsimony and interpretability of the solutions. To account for non-normally distributed data, we used the Maximum Likelihood estimator (MLR). Of the sample, 97% participated at T2 and 92% at T3. Bonferroni-corrected attrition analysis (*p* = 0.0028) indicated no significant differences in T1 variables between participants with missing data at least one measurement point and remaining participants, suggesting that data were Missing at Random (MAR; Little and Rubin, 2019). Therefore, missing data was accounted for by the Full Information Maximum Likelihood (FIML) procedure (Supplementary Table S2).

To test Hypothesis 3, we, first, conducted multinomial logistic regressions (MNLR). Here, we considered all SR competences simultaneously, thereby controlling for their respective influences, and accounted for the assignment of participants to trajectories by the expectation maximization (EM) algorithm (Muthén and Muthén, 2017). We used the R3Step procedure (Asparouhov and Muthén, 2014). Because this procedure uses list-wise deletion to handle missing data in the control variables that are not accounted for by FIML, missing values were accounted for by 20-times multiple imputation. We included all SR competences, gender, age, and SES as predictors of the likelihood to show the low AAB trajectory as the reference category as compared to showing the other three trajectories, respectively. MNLR is the state-of-the-art analysis that allows for statements about predictive effects that are important for the developmental aspect of our research questions (How do baseline SR competences predict AAB-trajectory membership?). However, it is limited by only allowing for conclusions about the relevance of SR competences for belonging to a certain trajectory in comparison to a single reference trajectory at a time. This limits the applicability of findings (e.g., for teachers to assess preventive needs of a single child based on results that require comparison to an unknown baseline group).

To address these issue, to account for potential rater (children, teachers, parents) and method (questionnaires, experimental games) effects, we, second, supplemented the MNLR analyses by ANOVAs based on the 20-times imputed data set in line with previous proceedings (Jung and Wickrama, 2008). This allowed us to compare all trajectories at one time without the constraint of a reference class as well as result patterns within, between, and across AAB measures. Results are also better suited for communication to practitioners. We estimated separate univariate linear models for each dependent SR variable within each imputed dataset. We pooled model estimates using Rubin's rules, and tested overall group effects with the pooled D1 *F*-test (Grund et al., 2016). We applied False Discovery Rate correction to *p*-values, followed by Holm-adjusted *post-hoc* contrasts. We computed effect sizes (η_*p*_^2^) per imputed dataset and averaged across imputations (van Ginkel, 2019). Finally, we visualized profiles of the *z*-standardized means of the SR competences and demographic variables to visualize SR-profiles per trajectory. We pre-registered our proceedings and hypotheses at: https://osf.io/thnf9 (Supplementary Material S12 for deviations).

## Results

### Descriptive statistics

Table 1 shows the descriptive statistics of all study variables. Bonferroni-corrected ANOVAs indicated more physical, proactive, and reactive aggression and total AAB in boys than in girls at all measurement points, and more NA-AB at T1. Girls showed higher T1 parent-rated inhibition, flexibility, planning, as well as lower Stroop interference, affective decision-making, and HRV than boys.

All AAB measures were positively correlated across measurement points (Supplementary Table S3 for details). Most SR competences were significantly correlated; HRV was only related to Stroop interference (Supplementary Table S4 for details). Supporting Hypothesis 1, parent-rated inhibition, flexibility, and planning were negatively related to all AAB measures at all measurement points; emotional reactivity was positively related to all AAB measures at all measurement points and Stroop interference was positively related to all AAB measures at nearly all measurement points. Updating and emotion regulation were negatively related to all outcomes at least one measurement point. Contrasting Hypothesis 1, affective decision-making, delay of gratification, and HRV were unrelated to almost all AAB measures (Table 2 for details).

**Table 2:** Correlations between the aggressive- and antisocial-behavior measures and the self-regulation competences for the total group.

Aggression and NA AB Measures	Working-Memory Updating	Stroop Inter-ference	Parent-Rated Inhibition	Flexibility	Emotional Reactivity	Emotion Regulation	Planning	Affective Decision-Making	Delay of Gratifi-cation	Heart Rate Variability	Gender	Age	SES
PhyA												
T1	**−0.102** ^***^	**0.108** ^***^	**−0.280** ^***^	**−0.202** ^***^	**0.189** ^***^	**−0.077** ^*^	**−0.407** ^***^	0.045	−0.019	0.026	**0.365** ^***^	−0.031	**−0.100** ^***^
T2	**−0.083** ^**^	**0.122** ^***^	**−0.278** ^***^	**−0.189** ^***^	**0.227** ^***^	**−0.088** ^**^	**−0.364** ^***^	0.036	0.025	0.020	**0.391** ^***^	−0.035	**−0.105** ^**^
T3	**−0.067** ^*^	**0.133** ^***^	**−0.202** ^***^	**−0.177** ^***^	**0.155** ^***^	−0.063	**−0.308** ^***^	**0.067** ^*^	−0.052	0.050	**0.316** ^***^	−0.015	**−0.121** ^***^
RelA												
T1	**−0.069** ^*^	**0.056** ^*^	**−0.246** ^***^	**−0.136** ^***^	**0.181** ^***^	**−0.067** ^*^	**−0.285** ^***^	−0.015	−0.022	0.020	0.044	−0.048	**−0.076** ^*^
T2	−0.044	0.041	**−0.172** ^***^	**−0.088** ^**^	**0.147** ^***^	**−0.072** ^*^	**−0.225** ^***^	−0.031	−0.003	−0.015	**0.065** ^*^	−0.039	**–**0.055
T3	**−0.115** ^***^	**0.093** ^**^	**−0.176** ^***^	**−0.167** ^***^	**0.113** ^***^	−0.016	**−0.218** ^***^	−0.011	−0.051	**0.076** ^*^	0.042	−0.011	**−0.155** ^***^
ProA												
T1	−0.048	0.044	**−0.236** ^***^	**−0.129** ^***^	**0.140** ^***^	**−0.066** ^*^	**−0.342** ^***^	0.014	−0.012	**0.061** ^*^	**0.228** ^***^	−0.031	−0.053
T2	−0.058	**0.086** ^**^	**−0.237** ^***^	**−0.102** ^***^	**0.144** ^***^	−0.058	**−0.308** ^***^	−0.004	0.032	0.018	**0.206** ^***^	0.006	**−0.100** ^**^
T3	**−0.097** ^**^	**0.068** ^*^	**−0.187** ^***^	**−0.129** ^**^	**0.099** ^**^	0.006	**−0.225** ^***^	0.017	0.021	0.055	**0.204** ^***^	−0.004	**−0.146** ^***^
ReaA												
T1	**−0.058** ^*^	**0.104** ^***^	**−0.237** ^***^	**−0.162** ^***^	**0.186** ^***^	**−0.111** ^***^	**−0.372** ^***^	0.022	0.00	0.021	**0.235** ^***^	**−0.066** ^*^	**−0.060** ^*^
T2	−0.048	**0.119** ^***^	**−0.265** ^***^	**−0.134** ^***^	**0.248** ^***^	**−0.135** ^***^	**−0.329** ^***^	0.026	**0.023** ^*^	0.013	**0.279** ^***^	−0.002	−0.044
T3	**−0.116** ^***^	**0.144** ^***^	**−0.189** ^***^	**−0.206** ^***^	**0.131** ^***^	−0.040	**−0.278** ^***^	−0.003	−0.076	−0.006	**0.176** ^***^	**−0.079** ^*^	**−0.137** ^***^
NA												
AB												
T1	**−0.070** ^*^	**0.119** ^***^	**−0.368** ^***^	**−0.118** ^***^	**0.353** ^***^	−0.050	**−0.288** ^***^	0.014	−0.013	−0.013	**0.108** ^***^	−0.015	**−0.181** ^***^
T2	**−0.079** ^**^	**0.134** ^***^	**−0.288** ^***^	**−0.124** ^***^	**0.283** ^***^	−0.058	**−0.235** ^***^	−0.016	−0.034	0.022	0.042	−0.043	**−0.121** ^***^
T3	**–**0.049	**0.063** ^*^	**−0.290** ^***^	**−0.063** ^*^	**0.271** ^***^	**−0.090** ^**^	**−0.233** ^***^	0.021	−0.005	0.001	**0.083** ^**^	−0.015	**−0.122** ^***^
AAB												
T1	**−0.08** ^**^	**0.117** ^***^	**−0.361** ^***^	**−0.173** ^***^	**0.281** ^***^	**−0.096** ^**^	**−0.445** ^***^	0.009	−0.020	0.025	**0.240** ^***^	**−0.070** ^*^	**−0.124** ^***^
T2	**−0.091** ^*^	**0.14** ^***^	**−0.290** ^***^	**−0.166** ^***^	**0.241** ^***^	**−0.092** ^*^	**−0.377** ^***^	−0.003	−0.012	−0.004	**0.267** ^***^	−0.044	−0.052
T3	**−0.108** ^**^	**0.127** ^***^	**−0.277** ^***^	**−0.178** ^***^	**0.170** ^***^	−0.073	**−0.307** ^***^	0.050	−0.039	0.046	**0.206** ^***^	−0.002	**−0.129** ^**^

^*^ *p* < 0.05, ^**^ *p* < 0.01, ^***^ *p* < 0.001; A, aggression; Phy, physical; Rel, relational; Pro, proactive; Rea, reactive; NA AB, non-aggressive antisocial behavior; AAB, total AAB score; SES, socio-economic status. Significant correlation coefficients are highlighted in bold-type.

### Trajectories of aggressive and antisocial behavior

To test Hypothesis 2, we examined models with 1–5 latent trajectories for all AAB measures in our study to determine the number of latent trajectories. For both, physical and relations aggression, the fit statistics and interpretability of findings suggested four-trajectory solutions based on significant VLMRT and substantial reductions in AIC, BIC, and aBIC compared to the three-trajectory, but not the five-trajectory solution, respectively (Supplementary Table S5 for details). For proactive aggression, fit statistics supported the five-trajectory solution, but inspections of trajectories demonstrated that a fifth decreasing trajectory did not add to interpretability. Similarly, for reactive aggression, a further fifth parallel trajectory did not add to interpretability. For matters of parsimony, interpretability, and comparability, we selected the four-trajectory solution in both cases. For NA-AB, VLMRT suggested the three-trajectory solution, whereas the other fit-indices also supported the four-trajectory solution. For the total AAB score, fit indices supported the four-trajectory solution.

For physical and reactive aggression, there were stable low, increasing, decreasing, and stable high trajectories, respectively. For relational and proactive aggression as well as the total AAB score, there were low, moderate low, moderate high, and high trajectories mainly with decreasing trends, respectively. For NA-AB, there were low, moderate low, increasing and decreasing trajectories. Group sizes varied between 2.2 and 81.8% of all participants and mostly between 10 and 63% (Figure 1; Supplementary Table S6 for intercept and slope means).

**Figure 1:**
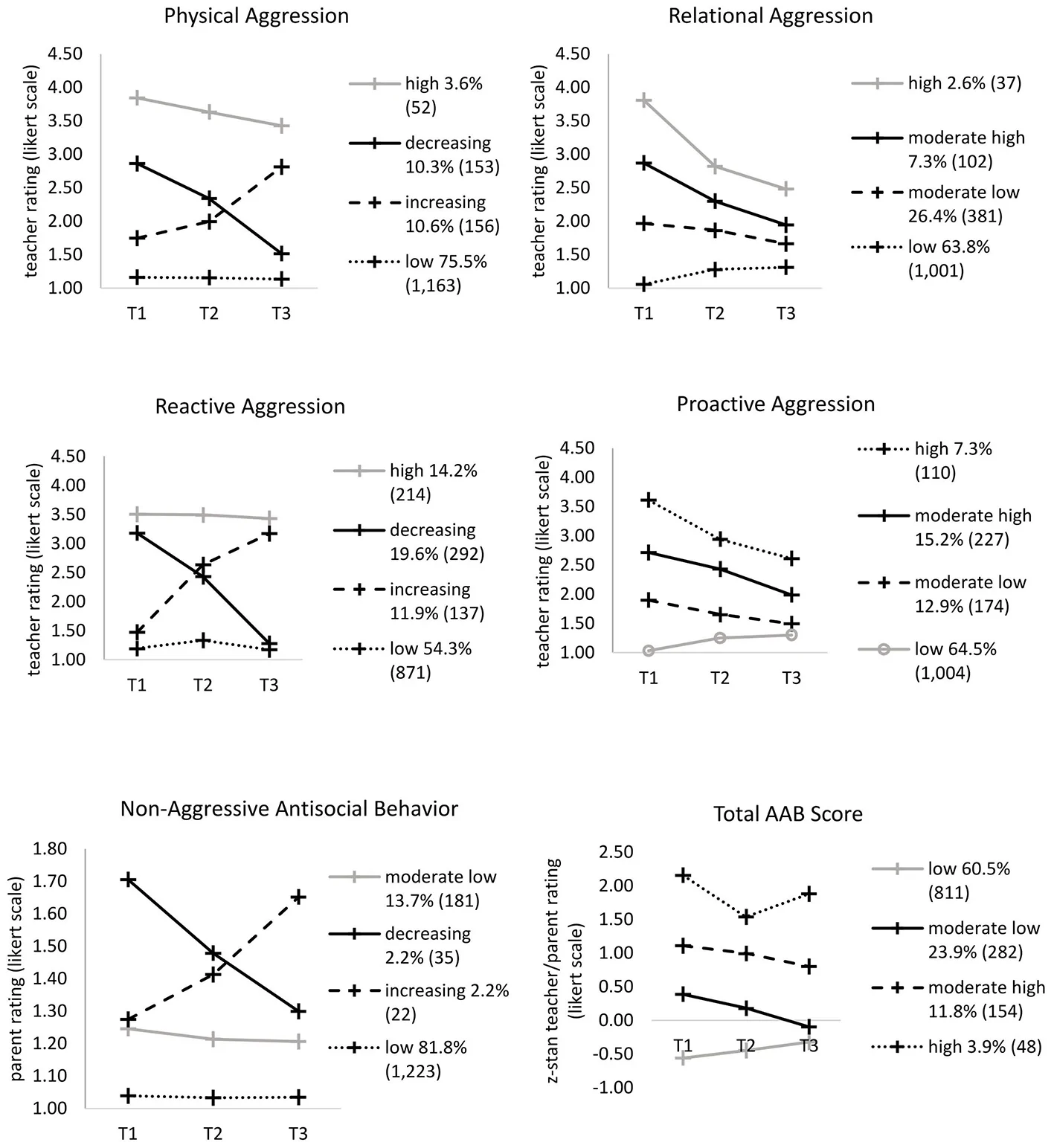
Trajectories of aggressive and antisocial behavior (AAB) across three time points. Percentages refer to the percentage of the respective sample (%) that had the highest probability of belonging to the respective trajectory. *N* of children in each class in brackets. Total AAB score from *z*-standardized means of single scales. *N*_physical aggression_ = 1,524; *N*_relational aggression_ = 1,521; *N*_proactive aggression_ = 1,515; *N*_reactive aggression_ = 1,514; *N*_antisocial behavior_ = 1,461; *N*_AAB_ = 1,295; range_aggression measures_ = 1–5; range_antisocial behavior_ = 1–3.

MANOVAs showed overall trajectory effects for all AAB measures. Physical- and proactive-aggression trajectories significantly differed in mean levels at all three measurement points; for the other four AAB measures, there were only single exceptions of no differences between trajectories (Supplementary Table S7 for details).

### Predictions of trajectory membership by all T1 SR competences

We then examined whether SR competences at T1 predicted the probability of belonging to a certain trajectory when compared to the low trajectory as the reference class. Figure 2 shows the pattern of mean values of the *z*-standardized SR competences per AAB measure and trajectory. Regarding *physical aggression*, MNLR including *N* = 1,524 children indicated that children on the low trajectory showed higher parent-rated inhibition and planning, and were more often girls than children on the other three trajectories (Supplementary Table S8 for details). In addition, they showed higher flexibility than children on the decreasing trajectory.

**Figure 2:**
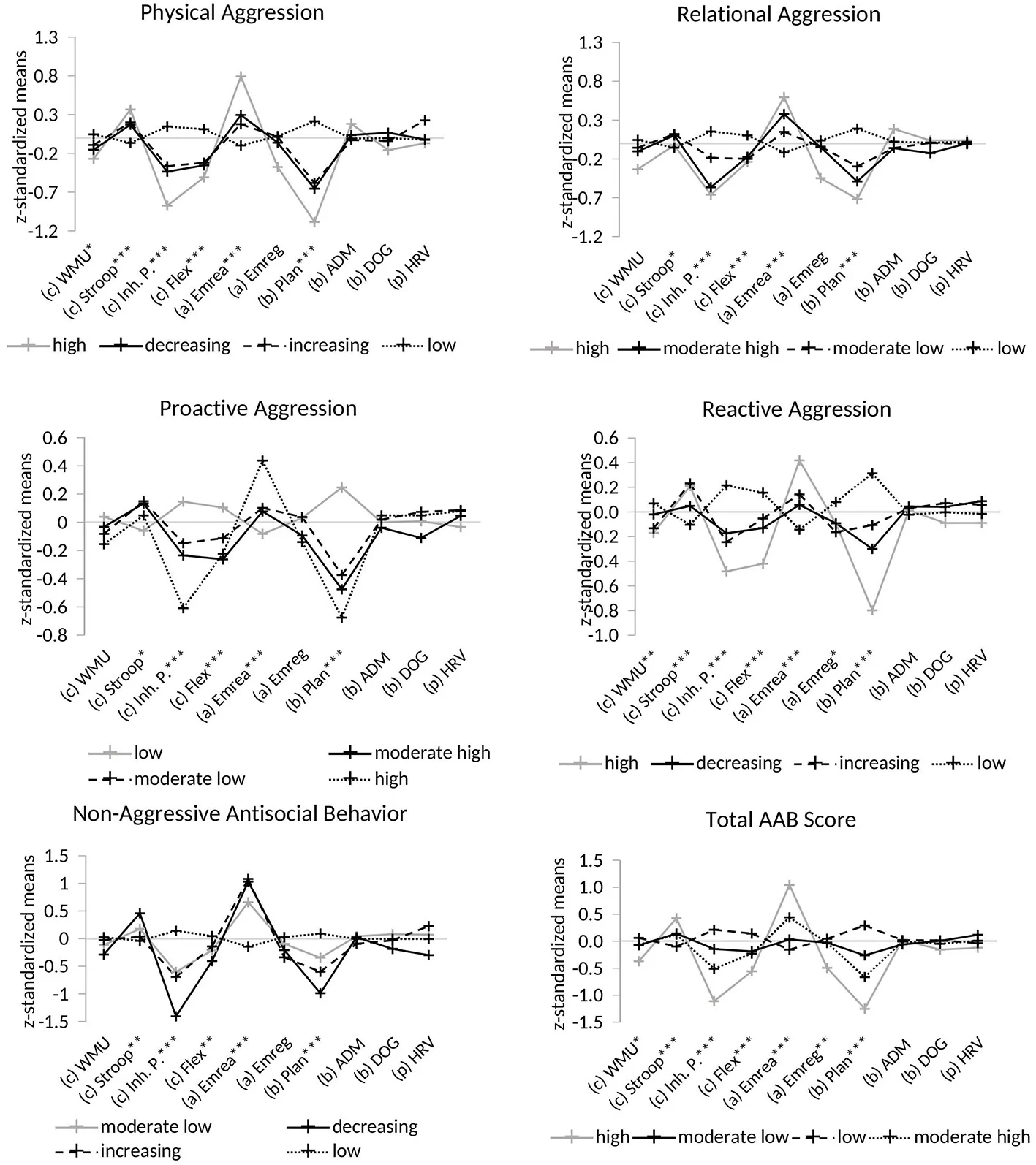
Z-standardized means of self-regulatory competences across trajectories of forms and functions of aggression as well as antisocial behavior. Flex, flexibility; Inh. p., parent-rated inhibition; Stroop, stroop interference; WMU, working-memory updating; Plan, planning; Emrea, emotional reactivity; Emreg, emotion regulation; ADM, affective decision making; DOG, delay of gratification; HRV, heart rate variability. Significant overall group effects in ANOVAs: *p < 0.05, **p < 0.01, ***p < 0.001.

Regarding *relational aggression*, MNLR including *N* = 1,521 children indicated that children on the low trajectory showed higher parent-rated inhibition and planning than children on the other three trajectories. Additionally, they showed higher flexibility and were older than children on the moderate low trajectory (Supplementary Table S8).

Regarding *proactive aggression*, MNLR including *N* = 1,515 children indicated that children on the low trajectory showed higher planning than children on the other three trajectories, higher parent-rated inhibition than children on the high and moderate high trajectories, higher Stroop interference than children on the high trajectory, higher flexibility and lower updating than children on the moderate high trajectory, and that they were more often girls than children on the high and the moderate high trajectories (Supplementary Table S9).

Regarding *reactive aggression*, MNLR including *N* = 1,514 children indicated that children on the low trajectory showed higher planning and parent-rated inhibition, and were more often girls than children on all other three trajectories. In addition, they showed higher flexibility and lower emotional reactivity than children on the high trajectory, and were older than children on the increasing trajectory (Supplementary Table S9).

Regarding NA-AB, MNLR including *N* = 1,461 children indicated that children on the low trajectory showed lower emotional reactivity than children on all other three trajectories, higher parent-rated inhibition than children on the moderate low and decreasing trajectories, and higher planning than children on the increasing and decreasing trajectories. In addition, they showed higher SES than children on the moderate low trajectory (Supplementary Table S9).

Regarding the *total AAB score*, MNLR including *N* = 1,295 children indicated that children on the low trajectory showed higher parent-rated inhibition and planning, and were more often girls than children on all other three trajectories. They showed lower emotional reactivity than children on the high and moderate high trajectory and were older than children on the moderate low trajectory (Supplementary Table S8).

Complementing the MNLR results, pooled ANOVAs without reference-group constraints revealed the additional relevance of some SR measures as well as more fine-grained mean-level differences when simultaneously comparing all trajectories (Supplementary Materials S10, S11). Across all AAB measures, children on the stable low trajectories not only showed consistently higher inhibition, planning, and flexibility; but also lower emotional reactivity and higher Stroop interference than children on the three other trajectories. Particularly planning, inhibition, and emotional reactivity were relevant for more group differences between the other three trajectories of most AAB measures. This pattern was most pronounced for the total AAB score with significant differences between all trajectories for, and clearly decreasing patterns in parent-rated inhibition, planning, and increasing in emotional reactivity for increasing AAB (low </> moderate low </> moderate high </> high).

## Discussion

This study aimed to extend the present research on the links between forms and functions of aggression as well as non-aggressive antisocial behavior (NA-AB) and multiple self-regulatory (SR) competences from middle childhood into early adolescence. Covering a highly relevant age range in a large sample and three measurement points, findings detail the similarities and differences in SR patterns associated with longitudinal AAB development. Most SR competences in our study (excluding *behavioral* affective decision making, delay of gratification, and *physiological* HRV) tended to show the expected associations with the AAB measures and trajectories even when simultaneously considered with higher SR related to lower AAB. We identified four trajectories for six AAB measures: high, increasing, decreasing, and low trajectories for physical and reactive aggression; increasing, decreasing, moderate low, and low trajectories for NA-AB; as well as high, moderate high, moderate low, and low trajectories with decreasing progressions for relational and proactive aggression as well as the total AAB score. Across measures, children on the low trajectories showed better SR competences than those on the three other trajectories, most consistently in parent-rated *cognitive* inhibition and *behavioral* planning abilities, but also in *cognitive* Stroop interference and flexibility, as well as *affective* emotional reactivity and emotion regulation. Thus, within AAB measures, sometimes pronounced, sometimes subtle differences in SR competences were associated with differential long-term tendencies to engage in or refrain from AAB. In contrast, trajectories and SR patterns across AAB measures tended to show strong similarities, suggesting that general intervention measures are justified and applicable whenever levels of AAB are elevated, irrespective of the AAB subtype displayed.

### Links between SR competences and AAB

In line with previous research and Hypothesis 1 (Ersan, 2020; O'Toole et al., 2019; Poland et al., 2016; Rohlf et al., 2017; White et al., 2013), we found overall negative correlations between higher SR competences and AAB, supporting the reliability of our results and suggesting that previous findings also apply to community samples in middle childhood and early adolescence. There were similar correlation patterns across the AAB measures in our study including proactive aggression. This contrasted pertinent theoretical assumptions, but supported previous empirical findings.

Nearly no relations with affective decision-making supported some previous research (Poland et al., 2016). Contrasting previous research (O'Toole et al., 2019; Subramani, 2020), delay of gratification was unrelated to most AAB measures, presumably due to ceiling effects and low variance in our measure. Considering HRV, our findings support previous research that indicated no or inconsistent associations with AAB (Jankovic et al., 2021).

### Trajectories of AAB

Identifying four low, increasing, decreasing, and high trajectories for physical and reactive aggression confirmed Hypothesis 2 (at least two trajectories per AAB measure) and resonated with previous research on forms of aggression that had often identified three or four similar trajectories (Côté et al., 2007; Cui et al., 2016). Four fairly parallel, often decreasing trajectories for relational and proactive aggression as well as the total AAB score suggested a general decrease in these behaviors from middle childhood to early adolescence irrespective of its relative level. Four low, moderate low, increasing, and decreasing trajectories for NA-AB tended to more strongly resemble findings on general antisocial behavior (Perry et al., 2018; Roisman et al., 2010) than on NA-AB specifically, due to the absence of a high and the presence of a decreasing trajectory (Givens and Reid, 2019; van Lier et al., 2009). The occurrence of a moderate low instead of a high and the presence of a chronic low NA-AB trajectory are consistent with findings that AB is rare in child community samples, whereas the decreasing trajectories aligns with findings of childhood-limited antisocial behavior (Burt, 2012; Givens and Reid, 2019; Maughan et al., 2000; Moffitt, 2015). Importantly, despite overall low mean scores, the present four trajectories suggest early developmental heterogeneity in NA-AB.

Note that LCGA favors four-class solutions and thereby potentially overestimates similarities between outcome measures in the present and with findings of previous studies (McNeish et al., 2023). We, however, found distinct trajectory patterns across AAB facets, supporting the validity of our findings. Analyses including the total AAB score underlined the additional information gain from investigating the single facets by showing diverging trajectory patterns for physical and reactive aggression as well as NA-AB.

Stable high and low AAB trajectories across middle childhood into early adolescence comprised 64% (total AAB) to 79% (physical aggression) and therewith the majority of the respective subsamples. But substantial groups of children showed marked changes in AAB in this age range, particularly regarding relational and reactive aggression and total AAB. This suggests a behavioral malleability that offers both, opportunities and need for intervention. Significant differences in AAB and SR mean values between trajectories at T1 suggest that children with potentially maladaptive long-term behavior can be identified early on and administered to interventions. Of note, mean values of children particularly on the high-aggression trajectories were roughly three times higher than for children on the respective low trajectories at this time point. The present findings strongly indicate that whenever children show only slightly elevated levels of AAB, pre- and intervention seems advisable irrespective of the individual SR patterns, because AAB levels tended to remain higher than those of the lowest group over time, even for children in the decreasing groups. However, given the overall low mean values of the present AAB measures, the high trajectories are mainly to be understood in reference to the present community sample and should be considered as at-risk trajectories, but do not necessarily indicate clinical relevance.

With 54%−82% of the sample, the low trajectories comprised the largest proportions of children in our sample for all AAB measures; all other trajectories comprised less than 27% of the sample, respectively. Thus, consistently showing no or hardly any AAB, that is, aggressive behavior or NA-AB, seems the norm in middle childhood and early adolescence (Evans et al., 2021; Fernandez Castelao and Kröner-Herwig, 2014; Roisman et al., 2010). Consequently, even children starting with only slightly higher AAB values than those in the low groups at T1 or ending at only slightly higher values than those in the low groups at T3 should still be considered as being on potentially maladaptive developmental pathways requiring early pre- and intervention to avoid chronic AAB with later detrimental psychosocial outcomes (Odgers et al., 2008).

### Differences of self-regulatory competences between trajectories of AAB

Overall, we found consistent differences in SR competences between trajectories within AAB measures, but rather overlapping patterns of trajectories and SR competences between AAB measures. In line with Hypothesis 3, children on the different AAB trajectories also differed in their SR competences at T1, and SR competences at T1 differentially predicted the probability of belonging to a certain trajectory group in reference to the low group. Thus, the present study extends previous research by showing that these competences are important associates not only of AAB in general, but also of its different trajectories. Thus, except for affective decision making, our findings support previous theoretical assumptions on the relevance of SR (Crick and Dodge, 1994; Moffitt, 1993).

Overall, children on the low AAB trajectories showed consistently higher SR competences than all other groups, particularly than those on the high trajectories. SR competences with the strongest overall effects in some cases even showed differences between children on the other three trajectories—particularly between the high trajectory and the increasing/moderate high and decreasing/moderate low trajectories. Hence, within and across all six AAB measures, the present findings support the general idea of central SR aspects being underdeveloped in children with higher AAB levels and that SR competences generally protect from higher AAB. Even proactive aggression followed this pattern, although variable-centered research had often indicated no relations with many SR measures. This underscores that person-centered analyses may further a more fine-grained understanding of differential relations between different AAB measures and SR competences. Negligible differences between children on the intermediate trajectories (increasing/decreasing, moderate high/low) indicate that—against expectations—, the specific elevated AAB trajectory is hardly predictable by SR competences, underlining that all children with initially higher AAB levels (hence, notable or more frequent AAB in relation to peers) may profit from early general prevention instead of tailored later interventions.

Overall, MNLR analysis and ANOVAs yielded similar results and reflected the correlation results. However, ANOVA results extended MNLR findings and provided finer-grained differences, particularly for proactive aggression, and highlighted the relevance of questionnaire-based parent- and teacher ratings, but also the experimentally assessed SR competences. The links between the AAB and SR measures seemed robust (e.g., were shown for both, experimentally assessed Stroop interference as well as parent-rated inhibition, whereas the aggression measures were teacher-rated), underlining their validity.

Parent-rated *cognitive* inhibition and *behavioral* planning consistently predicted the probability of belonging to a certain trajectory group in reference to the low group and differentiated between children on the low and children on all other trajectories across all six AAB measures. This suggests a transdiagnostic relevance of the abilities to suppress primary impulses and to develop and follow up on long-term plans for lower AAB in middle childhood and early adolescence. Both these SR competences highlight the importance of prioritizing long-term benefits over short-term interests and according behavior modulation.

ANOVA results additionally suggested the relevance of *cognitive* flexibility and *affective* emotional reactivity for all AAB measures and emotion regulation for the total AAB score. As suggested by the information processing model, flexibility may be important in producing a variety of potential, also non-aggressive responses to difficult social situations (Crick and Dodge, 1996). Dealing with frequent and intense emotions as a consequence of anger reactivity may require resources that cannot be used for controlling one's behavior (Gabel and McAuley, 2020). Thus, a lower tendency to experience negative emotions as well as the ability to modulate these emotions are likely to prevent from AAB.

Overall, these findings add to the current state of SR research by suggesting that certain SR competences may be more central for low AAB trajectories than others. Thus, it does not seem necessary to address all SR competences when tackling AAB.

### Differential findings between AAB measures

Despite predominant overlaps in findings across AAB measures, some noteworthy differences emerged and further underlined the utility of the person-centered approach. Regarding forms of aggression, the stronger effects of *cognitive* Stroop interference and *behavioral* planning for physical than relational aggression suggest a stronger relevance of (dys)regulated impulsivity for physical aggression; in contrast, smaller effects in relational than in physical aggression, particularly of planning underline a requirement for (complex) SR skills enabling social understanding to perform relational aggression (Crick and Grotpeter, 1995). In line with previous research, being a boy predicted physical aggression, but was negligible for relational aggression (Card et al., 2008), whereas higher age at T1 was negligible for physical aggression that already peaks in early childhood, but predicted relational aggression that increases throughout childhood and into adolescence (Casas and Bower, 2018).

Regarding functions of aggression, low inhibition and planning were more pronounced in reactive than in proactive aggression, indicating that proactive aggression requires more SR competences. This assumption is further supported by MNLR findings showing that lower *cognitive* Stroop interference predicted a higher probability of belonging to the high than the low proactive aggression trajectory, and higher *cognitive* updating predicted a higher probability of belonging to the moderate high than the low proactive aggression trajectory. However, inconsistencies between MNLR and ANOVA results and positive correlations of Stroop interference with T2 and T3 proactive aggression and a negative correlation of updating and T3 proactive aggression might also indicate spurious or suppressor effects when simultaneously testing effects of SR competences against each other. In addition, MNLR findings related emotional reactivity, and ANOVA results related emotion regulation particularly to reactive aggression, supporting the notion of particularly low *affective* SR among children high in reactive aggression (Crick and Dodge, 1996; Vitaro et al., 2006).

Finally, for NA-AB gender differences showed the highest effect size after planning, confirming that it is a key predictor of diverging developmental trajectories (Moffitt, 2018).

## Limitations and outlook

The strengths of the current study include using longitudinal multi-method data from a large community sample with high retention rates, considering a large set of diverse AAB measures and SR competences, and using different statistical procedures to gain robust results on the links between the AAB measures and their trajectories and the SR competences. Limitations include low internal consistencies of some measures that may limit the generalizability of related findings. They most likely reflect the independence of different emotion-regulation strategies and of different aspects of NA-AB, floor and ceiling effects in NA-AB and delay of gratification, as well as rater- and method effects (e.g., internal consistencies tend to be lower in children than older samples or in experimental tasks than in questionnaire due to state influences (Duckworth and Kern, 2011). Fixing proactive and reactive aggression to zero for participants for which no relational or physical aggression was reported, may have obscured aggressive behavior for other reasons. Because children's SR-competences were not measured prior to the AAB measures, we cannot draw strong causal inferences about their influence on the AAB trajectories. It was beyond the scope of the present study to account for further SR-competences and external influences that are also relevant for AAB, such as sensation seeking, behavioral control, or family risk factors. Due to space limitations and the complexity of results, we decided against permutating through all possible trajectory combinations for a comprehensive comparison about differential predictions in MNLR. Finally, because our community sample showed floor effects in all AAB measures, trajectory groups should not be considered as distinct clinical categories, but rather be interpreted as empirical patterns indicating how fostering SR competences might support lower AAB trajectories over time. Future research may concentrate on inconsistent effects regarding proactive aggression, use more reliable measures of some and consider further SR competences, potential gender differences, as well as additional measurement points that allow for modeling non-linear trajectories. It may consider interrelations of the AAB measures in AAB profiles and further AAB correlates, such as social factors, to clarify the relative importance of SR competences when predicting AAB.

## Conclusion

The current study largely confirmed negative associations between a broad range of SR competences and forms and functions of aggression as well as NA-AB, largely irrespective of the AAB measure. This strongly suggests that SR competences are important in protecting from AAB in general. Four distinct trajectories across all six AAB measures indicated developmental similarities in long-term tendencies to show different AAB. Inhibition and planning were most consistently associated with and may be the most central SR competences to protect from any type of AAB and to be addressed by prevention and intervention measures. In addition, flexibility as well as emotional reactivity were relevant for all AAB measures. These findings suggest that similar interventions that address a broad range of SR competences may protect from AAB as a whole. Because middle childhood to early adolescence is a vulnerable phase for the onset of AAB and because the present study showed early links with SR competences, interventions should be designed for this age group. This can for example be achieved by establishing specific training programs for children that display early problem behavior in terms of selective prevention, but also by universal prevention efforts, such as actively promoting planning skills in school.

## Data Availability

The data analyzed in this study is subject to the following licenses/restrictions: The data are part of a multi-site research consortium and are not publicly available due to data protection and governance regulations. Analysis code and study materials can be granted and data can be granted upon reasonable request. Requests to access these datasets should be directed to Rebecca Bondü, r.bondue@phb.de.
