# Supplementary material for: Links between self-regulation competences and aggressive and non-aggressive antisocial behavior in middle childhood and early adolescence: a three-wave longitudinal study
Source: Front Psychol. 2026 Sep 23;17:1866377. doi: 10.3389/fpsyg.2026.1866377 (PMC13642200; doi:10.3389/fpsyg.2026.1866377)
Supplement: Supplementary file 1 [file Data_Sheet_1.pdf]

Supplementary Material

Table S1

Expected Positive and Negative Associations of SR Competences and Physical, Relational, Proactive, Reactive Aggression and Non-Aggressive Antisocial Behavior

Type of SR competence	SR competence	Expected positive associations with	Expected negative associations with
Cognitive	Working memory updating		Physical aggression<br>Relational aggression<br>Reactive aggression<br>Non-aggressive antisocial behavior
	Inhibition		Physical aggression<br>Relational aggression<br>Reactive aggression<br>Non-aggressive antisocial behavior
	Flexibility		Physical aggression<br>Relational aggression<br>Reactive aggression<br>Non-aggressive antisocial behavior
Affective	Emotional reactivity	Physical aggression<br>Relational aggression<br>Reactive aggression<br>Non-aggressive antisocial behavior	
	Emotion regulation		Physical aggression<br>Relational aggression<br>Reactive aggression<br>Non-aggressive antisocial behavior
Behavioral	Planning		Physical aggression<br>Relational aggression<br>Reactive aggression<br>Non-aggressive antisocial behavior
	Affective decision making	Non-aggressive antisocial behavior	
	Delay of gratification		Physical aggression<br>Relational aggression<br>Reactive aggression<br>Non-aggressive antisocial behavior
Physiological	Heart rate variability	Relational aggression<br>Non-aggressive antisocial behavior	(Physical aggression)<br>Reactive aggression<br>Non-aggressive antisocial behavior

Table S2

Attrition Analysis Comparing Baseline (T1) Characteristics of Participants Who Remained in the Study versus Those Who Dropped Out at T2 or T3 utilizing Chi-Square Test (Gender) and Independent Samples T-Tests

Variable	Mean Completers	Mean Dropouts	χ^2/ *t* statistic	df	*p* value
T1 Aggression					
Physical	1.48	1.69	-1.96	111.09	0.052
Relational	1.49	1.65	-2.01	114.1	0.047
Proactive	1.58	1.74	-1.62	115.7	0.107
Reactive	1.93	2.08	-1.21	115.62	0.229
NAAB	1.08	1.1	-1.08	86.05	0.283
T1 SR Competences					
WMU	6.19	6.11	0.6	154.35	0.548
Stroop	24.91	25.44	-0.66	155.34	0.512
Parent-Rated Inhibition	3.54	3.45	1.25	88.69	0.216
Flexibility	15.44	14.83	1.28	150.66	0.201
Emotional. Reactivity	2.41	2.58	-2.36	87.72	0.021
Emotion Regulation	2.19	2.12	0.86	86.64	0.395
Planning	3.72	3.55	1.68	113.09	0.095
ADM	5.56	4.72	0.91	164.86	0.366
DoG	2.79	2.74	0.47	143.75	0.636
HRV	58.64	57.98	0.25	135.79	0.801
Age	8.35	8.42	-0.76	154.17	0.448
SES	5.07	4.74	2.43	87.3	0.017
Gender	1.48	1.40	2.93	1	0.087

Note. *$p<.05$, **$p<.01$, ***$p<.001$; NAAB=non-aggressive antisocial behavior; WMU=working-memory updating; Stroop=Stroop interference; ADM=affective decision making; DoG=delay of gratification; HRV=heart rate variability; SES=socio-economic status. Bonferroni-corrected significance threshold (p=.0028).

Table S3

Correlations of Forms and Functions of Aggression, Non-Aggressive Antisocial Behavior, a Total AAB Score and Demographic Variables

Variable	1	2	3	4	5	6	7	8	9	10	11	12	13	14	15	16	17	18	19	20
1 T1 Phy A																				
2 T2	**.714*****																			
3 T3	**.477*****	**.502*****																		
4 T1 Rel A	**.615*****	**.450*****	**.334*****																	
5 T2	**.450*****	**.658*****	**.306*****	**.533*****																
6 T3	**.311*****	**.345*****	**.659*****	**.356*****	**.378*****															
7 T1 Proa A	**.722*****	**.573*****	**.412*****	**.747*****	**.499*****	**.372*****														
8 T2	**.541*****	**.732*****	**.441*****	**.488*****	**.740*****	**.418*****	**.617*****													
9 T3	**.399*****	**.457*****	**.595*****	**.336*****	**.371*****	**.685*****	**.441*****	**.507*****												
10 T1 Rea A	**.656*****	**.483*****	**.337*****	**.623*****	**.392*****	**.232*****	**.647*****	**.443*****	**.286*****											
11 T2	**.506*****	**.645*****	**.389*****	**.401*****	**.627*****	**.320*****	**.466*****	**.627*****	**.373*****	**.543*****										
12 T3	**.389*****	**.468*****	**.600*****	**.295*****	**.364*****	**.542*****	**.359*****	**.438*****	**.610*****	**.310*****	**.457*****									
13 T1 NAAB	**.309*****	**.294*****	**.295*****	**.252*****	**.194*****	**.273*****	**.255*****	**.231*****	**.227*****	**.206*****	**.234*****	**.202*****								
14 T2	**.213*****	**.228*****	**.276*****	**.236*****	**.167*****	**.282*****	**.218*****	**.192*****	**.173*****	**.152*****	**.176*****	**.187*****	**.596*****							
15 T3	**.192*****	**.177*****	**.247*****	**.206*****	**.141*****	**.204*****	**.204*****	**.176*****	**.233*****	**.151*****	**.174*****	**.151*****	**.526*****	**.528*****						
16 T1 AAB	**.852*****	**.640*****	**.477*****	**.829*****	**.546*****	**.412*****	**.865*****	**.588*****	**.445*****	**.809*****	**.562*****	**.397*****	**.537*****	**.367*****	**.327*****					
17 T2	**.640*****	**.86*****	**.527*****	**.554*****	**.840*****	**.457*****	**.618*****	**.861*****	**.494*****	**.526*****	**.815*****	**.513*****	**.378*****	**.462*****	**.307*****	**.694*****				
18 T3	**.504*****	**.478*****	**.809*****	**.450*****	**.343*****	**.820*****	**.491*****	**.496*****	**.828*****	**.325*****	**.408*****	**.761*****	**.413*****	**.394*****	**.507*****	**.557*****	**.539*****			
19 Gender	**.365*****	**.391*****	**.316*****	.044	**.065***	.042	**.228*****	**.206*****	**.204*****	**.235*****	**.279*****	**.176*****	**.108*****	.042	**.083****	**.240*****	**.267*****	**.206*****		
20 Age	-.032	-.035	-.017	-.049	-.039	-.013	-.031	.006	-.004	**-.066***	-.002	**-.079****	-.015	-.043	-.015	**-.070***	-.044	-.002	.032	
21 SES	**-.100****	**-.105****	**-.121*****	**-.076***	-.055	**-.155*****	-.053	**-.100****	**-.146*****	**-.060***	-.044	**-.137*****	**-.181*****	**-.121*****	**-.122*****	**-.124*****	-.052	**-.129****	.008	**-.080****

Note. $^{*}p$<.05, $^{**}p$<.01, $^{***}p$<.001; A=aggression, Phy=physical; Rel=relational; Proa=proactive; Rea=reactive; NAAB=non-aggressive antisocial behavior; AAB=total aggression and NAAB score; SES=socio-economic status.

Table S4

Correlations between Self-Regulation Competences and Demographic Variables

	1	2	3	4	5	6	7	8	9	10	11	12
1 WMU												
2 Stroop Interference	**-.270*** **											
3 Parent-Rated Inhibition	**.099*** **	**-.113*** **										
4 Flexibility	**.353*** **	**-.337*** **	**.142*** **									
5 Emotional Reactivity	**-.100*** **	**.103*** **	**-.476*** **	**-.086** **								
6 Emotion Regulation	-.014	-.021	**.133*** **	-.005	**-.400*** **							
7 Planning	**.262*** **	**-.287*** **	**.296*** **	**.305*** **	**-.163*** **	-.012						
8 ADM	**.076** **	-.028	.017	**.087*** **	-.033	-.037	.012					
9 DoG	**.070** **	**-.059* **	.030	**.100*** **	-.034	-.017	**.070* **	.027				
10 HRV	.004	**.060* **	-.045	.007	.015	-.042	-.022	.027	.043			
11 Age	**.245*** **	**-.345*** **	.050	**.289*** **	.008	.021	.019	**.061* **	**.110*** **	.001		
12 SES	**.183*** **	**-.084** **	**.169*** **	**.174** **	**-.053* **	**-.103*** **	**.258*** **	**.064* **	.028	-.010	**-.080** **	
13 Gender	.019	**.082*** **	**-.131*** **	**-.143*** **	.050	**-.062* **	**-.213*** **	**.135*** **	**.072** **	**.106*** **	.031	.008

Note. *p<.05, **p<.01, ***p<.001; WMU=working-memory updating; ADM=Affective decision-making; DoG=delay of gratification; HRV=heart rate variability; SES=socio-economic status

Table S5

Latent Class Growth Analyses: Fit-Indices of Trajectory Classes for Physical, Relational, Proactive, and Reactive Aggression as well as Non-Aggressive Antisocial Behavior and a Total AAB Score

***N* classes**	**Physical Aggression**						**Relational Aggression**					
	AIC	BIC	aBIC	VLMRT	*pBLRT*	Entropy	AIC	BIC	aBIC	VLMRT	*pBLRT*	Entropy
**1**	8455.88	8482.52	8466.64	-	-	-	7861.25	7887.89	7872.01	-	-	-
**2**	6894.56	6937.19	6911.78	0.0000	0.000	0.927	6931.59	6974.21	6948.80	0.0000	0.0000	0.856
**3**	6353.65	6412.27	6377.32	0.0121	0.000	0.906	6417.07	6475.67	6440.72	0.0010	0.0000	0.899
**4**	5993.77	6068.38	6023.91	0.0002	0.000	0.915	5996.74	6071.32	6026.85	0.0000	0.0000	0.939
**5**	5811.51	5902.10	5848.10	0.1085	0.000	0.891	5829.43	5919.99	5865.99	0.4952	0.0000	0.924
	**Proactive Aggression**						**Reactive Aggression**					
	AIC	BIC	aBIC	VLMRT	*pBLRT*	Entropy	AIC	BIC	aBIC	VLMRT	*pBLRT*	Entropy
**1**	8981.84	9008.45	8992.57	-	-	-	10885.06	10911.67	10895.79	-	-	-
**2**	7517.09	7559.67	7534.26	0.0000	0.000	0.904	9916.60	9959.18	9933.77	0.0000	0.0000	0.839
**3**	7056.51	7115.07	7080.12	0.0000	0.000	0.903	9636.85	9695.40	9660.45	0.0001	0.0000	0.803
**4**	6639.05	6713.57	6669.10	0.0088	0.000	0.916	9263.68	9338.19	9293.72	0.0001	0.0000	0.808
**5**	6285.38	6375.87	6321.87	0.0008	0.000	0.940	9048.08	9138.56	9084.56	0.0002	0.0000	0.829
	**Non-Aggressive Antisocial Behavior**						**Total AAB Score**					
	AIC	BIC	aBIC	VLMRT	*pBLRT*	Entropy	AIC	BIC	aBIC	VLMRT	*pBLRT*	Entropy
**1**	-4497.86	-4471.42	-4487.31	-	-	-	5744.72	5770.55	5754.67	-	-	-
**2**	-5802.13	-5759.83	-5785.25	0.0003	0.000	0.957	4829.68	4871.01	4845.60	0.0000	0.0000	0.853
**3**	-6292.73	-6234.58	-6269.52	0.1452	0.000	0.929	4523.68	4580.51	4545.57	0.0767	0.0000	0.849
**4**	-6538.22	-6464.20	-6508.67	0.1171	0.000	0.915	4364.49	4436.82	4392.35	0.0428	0.0000	0.853
**5**	-7660.20	-7570.32	-7624.33	0.5692	0.000	0.959	4285.09	4372.91	4318.91	0.3174	0.0000	0.838

Note. BIC=Bayesian Information Criterion, AIC=Akaike information criterion, aBIC=sample-size adjusted Bayesian Information Criterion; pbLRT=parametric Bootstrap-Likelihood-Ratio-Test for differences; VLMRT=Vuong-Lo-Mendell-Rubin-Test *p*-value.

Table S6

Intercept and Slope Means for the Four-Class LCGA Models Across Aggressive and Non-Aggressive Antisocial Behavior (NAAB) Measures and a Total AAB Score

**AAB Measure**	**Class**	**Class Size (%)**	**Intercept Mean *(SE)***	**Intercept *p***	**Slope Mean *(SE)***	**Slope *p***
**Physical A**	*High*	3.6	3.84 (0.12)	0.000	-0.21 (0.11)	0.046
	Decreasing	10.3	2.89 (0.09)	0.000	-0.67 (0.06)	0.000
	Increasing	10.6	1.69 (0.07)	0.000	0.52 (0.07)	0.000
	Low	75.5	1.17 (0.012)	0.000	-0.01 (0.01)	0.075
**Relational A**	*High*	2.6	3.80 (0.08)	0.000	-0.75 (0.15)	0.000
	Moderate High	7.3	2.87 (0.03)	0.000	-0.49 (0.05)	0.000
	Moderate Low	26.4	1.97 (0.01)	0.000	-0.15 (0.02)	0.000
	Low	63.8	1.06 (0.01)	0.000	0.15 (0.01)	0.000
**Proactive A**	*High*	7.3	3.61 (0.04)	0.000	-0.55 (0.07)	0.000
	Moderate High	15.2	2.72 (0.05)	0.000	-0.37 (0.04)	0.000
	Moderate Low	12.9	1.91 (0.06)	0.000	-0.21 (0.05)	0.000
	Low	64.5	1.03 (0.00)	0.000	0.15 (0.01)	0.000
**Reactive A**	*High*	14.2	3.44 (0.07)	0.000	-0.02 (0.05)	0.675
	Decreasing	19.6	3.24 (0.07)	0.000	-0.97 (0.04)	0.000
	Increasing	14.2	1.48 (0.07)	0.000	0.86 (0.07)	0.000
	Low	54.3	1.21 (0.02)	0.000	-0.1 (0.01)	0.482
**NAAB**	*Decreasing*	2.2	1.71 (0.05)	0.000	-0.21 (0.03)	0.000
	Increasing	2.2	1.27 (0.04)	0.000	0.18 (0.04)	0.000
	Moderate Low	13.7	1.22 (0.02)	0.000	-0.02 (0.01)	0.13
	Low	81.8	1.04 (0.00)	0.000	-0.00 (0.00)	0.186
**Total AAB**	*High*	3.9	2.11 (0.10)	0.000	-0.27 (0.10)	0.006
	Moderate High	11.8	1.10 (0.08)	0.000	-0.18 (0.07)	0.010
	Moderate Low	23.9	0.39 (0.05)	0.000	-0.23 (0.04)	0.000
	Low	60.5	-0.55 (0.01)	0.000	0.11 (0.01)	0.000

Table S7

Differences between Trajectories in Means of Aggressive and Non-Aggressive Antisocial Behavior and a Total AAB Score between Three Measurement Points

	**Trajectories**					
	Low	***Increasing***	***Decreasing***	***High***		
	M (SD)	*M (SD)*	*M (SD)*	*M (SD)*	*F*	η_p^2
**Physical A**						
**T1**	1.16 (0.30)^{a}	1.76 (0.48)^{a}	2.87 (0.53)^{a}	3.87 (0.61)^{a}	832.68***	.754
**T2**	1.16 (0.33)^{a}	2.00 (0.66)^{a}	2.36 (0.71)^{a}	3.67 (0.87)^{a}	406.23***	.599
**T3**	1.14 (0.29)^{a}	2.83 (0.60)^{a}	1.49 (0.48)^{a}	3.45 (0.85)^{a}	772.65***	.740
**Reactive A**						
**T1**	1.19 (0.38)^{a}	1.47 (0.52)^{a}	3.18 (0.65)^{a}	3.50 (0.64)^{a}	995.72***	.797
**T2**	1.33 (0.65)^{ab}	2.63 (1.18)^{a}	2.43 (1.08)^{b}	3.49 (0.84)^{ab}	167.42***	.398
**T3**	1.17 (0.37)^{a}	3.17 (0.67)^{a}	1.28 (0.44)^{a}	3.43 (0.62)^{a}	949.36***	.789
	Low	***Moderate Low***	***Moderate High***	***High***		
**Relational A**						
**T1**	1.05 (0.12)^{a}	1.97 (0.23)^{a}	2.89 (0.21)^{a}	3.82 (0.28)^{a}	4607.86***	.947
**T2**	1.28 (0.53)^{abc}	1.88 (0.76)^{abd}	2.28 (0.95)^{b}	2.84 (0.99)^{cd}	84.17***	.244
**T3**	1.32 (0.56)^{abc}	1.69 (0.79)^{ad}	1.95 (0.74)^{b}	2.59 (1.05)^{cd}	35.63***	.120
**Proactive A**						
**T1**	1.03 (0.12)^{a}	1.90 (0.23)^{a}	2.71 (0.25)^{a}	3.61 (0.35)^{a}	4702.06***	.950
**T2**	1.26 (0.55)^{a}	1.65 (0.80)^{a}	2.43 (0.78)^{a}	2.94 (0.89)^{a}	157.68***	.390
**T3**	1.30 (0.63)^{a}	1.49 (0.80)^{a}	1.99 (0.97)^{a}	2.61 (1.10)^{a}	76.983***	.238
**Total AAB**						
**T1**	-0.56 (0.19)^{a}	0.39 (0.27)^{a}	1.11 (0.30)^{a}	2.16 (0.43)^{a}	943.92***	.880
**T2**	-0.45 (0.38)^{abc}	0.18 (0.59)^{abc}	0.99 (0.58)^{b}	1.54 (0.79)^{c}	136.04***	.513
**T3**	-0.33 (0.44)^{a}	-0.10 (0.56)^{a}	0.80 (0.61)^{a}	1.88 (0.62)^{a}	88.20***	.406
	Low	***Moderate Low***	***Increasing***	***Decreasing***		
**NAAB**						
**T1**	1.04 (0.07)^{abc}	1.23 (0.13)^{ac}	1.28 (0.18)^{bd}	1.69 (0.13)^{cd}	484.04***	.627
**T2**	1.03 (0.06)^{abc}	1.21 (0.13)^{ad}	1.40 (0.21)^{b}	1.45 (0.21)^{cd}	322.28***	.528
**T3**	1.03 (0.07)^{abc}	1.20 (0.12)^{ab}	1.65 (0.19)^{bd}	1.30 (0.15)^{cd}	391.97***	.576

Note: Multivariate analyses of variance: *p <05, ** p<.01, ***p<.001; A=aggression; NAAB=non-aggressive antisocial behavior; AAB=total aggression and NAAB score (z-standardized); Same letters in hyperscripts indicate differences between the respective trajectories in post-hoc analyses (Games-Howell)

MANOVAs showed overall trajectory effects for physical (F(9, 2,445)=232.8, p<.001, $\eta^2 p$=.46), relational (F(9, 2,343)=121.03, p<.001, $\eta^2 p$=.32), proactive (F(9, 2,223)=119.22, p<.001, $\eta^2 p$=.33), and reactive aggression (F(9, 2,283)=264.44, p<.001 $\eta^2 p$=.51), as well as antisocial-behavior (F(9, 2,595)= 151.732, p<.001 $\eta^2 p$=.35) and total AAB (F(9, 1,161)=66.58, p<.001, $\eta^2 p$=.34). Most trajectories significantly differed in their mean levels of the respective AAB facet at all three measurement points. There were no differences between means of the high and moderate high relational-aggression trajectory at T2 and T3, the moderate high and moderate low relational-aggression trajectory at T3, the increasing and decreasing reactive-aggression trajectories at T2, the moderate low and increasing antisocial-behavior trajectory at T1 and T2, the increasing and decreasing antisocial-behavior trajectory at T2, the moderate low and decreasing antisocial-behavior trajectory at T3, and the moderate high and high total AAB trajectory at T2.

Table S8

Parameter Estimates of Multinomial Logistic Regression Analyses for the Likelihood to belong the Mentioned Trajectory as Compared to the Low Trajectory for Physical and Relational Aggression as well as Total Aggressive and Non-Aggressive Antisocial Behavior

Predictor	**Physical Aggression**		**Relational Aggression**		**Total AAB**	
	B (SE)	*OR* (reversed *OR*)	*B (SE)*	*OR* (reversed *OR*)	*B (SE)*	*OR* (reversed *OR*)
	High		***High***		***High***	
Flexibility	-0.28 (0.17)	0.75 (1.33)	-0.14 (0.20)	0.87 (1.15)	-0.31 (0.19)	0.74 (1.36)
Parent-Rated Inhibition	-0.60 (0.23)**	0.55 (1.83)	-0.45 (0.23)*	0.64 (1.57)	-0.95 (0.25)***	0.39 (2.57)
Stroop Interferences	-0.08 (0.17)	0.92 (1.09)	-0.31 (0.22)	0.74 (1.36)	-0.02 (0.17)	0.98 (1.02)
WMU	0.12 (0.17)	1.13	-0.13 (0.19)	0.88 (1.13)	0.18 (0.20)	1.20
Planning	-1.16 (0.22)***	0.31 (3.18)	-0.84 (0.22)***	0.43 (2.31)	-1.48 (0.25)***	0.23 (4.38)
Emotional Reactivity	0.41 (0.22)	1.51	0.28 (0.23)	1.32	0.60 (0.20)**	1.82
Emotion Regulation	-0.17 (0.22)	0.85 (1.18)	-0.36 (0.26)	0.70 (1.43)	-0.34 (0.25)	0.71 (1.41)
ADM	0.15 (0.15)	1.17	0.23 (0.19)	1.26	0.06 (0.19)	1.06
DoG	-0.05 (0.16)	0.95 (1.05)	0.07 (0.20)	1.07	-0.01 (0.19)	0.99 (1.01)
HRV	-0.13 (0.20)	0.88 (1.13)	-0.03 (0.22)	0.97 (1.03)	-0.30 (0.26)	0.74 (1.35)
Age	-0.05 (0.20)	0.95 (1.05)	0.10 (0.21)	1.10	0.09 (0.20)	1.09
SES	-0.17 (0.17)	0.84 (1.19)	-0.01 (0.20)	0.99 (1.01)	-0.26 (0.20)	0.77 (1.30)
Gender	2.61 (0.54)***	13.57	-0.37 (0.38)	0.69 (1.45)	0.90 (0.42)*	2.45
	Increasing		***Moderate High***		***Moderate High***	
Flexibility	-0.18 (0.11)	0.84 (1.19)	-0.1 (0.12)	0.92 (1.10)	-0.09 (0.12)	0.91 (1.10)
Parent-Rated Inhibition	-0.27 (0.13)*	0.77 (1.31)	-0.52 (0.13)***	0.60 (1.68)	-0.42 (0.13)***	0.66 (1.52)
Stroop Interferences	-0.06 (0.10)	0.94 (1.06)	-0.12 (0.13)	0.89 (1.13)	-0.17 (0.12)	0.84 (1.19)
WMU	0.16 (0.10)	1.17	0.11 (0.12)	1.12	0.19 (0.12)	1.21
Planning	-0.71 (0.12)***	0.49 (2.03)	-0.65 (0.14)***	0.52 (1.92)	-1.03 (0.13)***	0.36 (2.81)
Emotional Reactivity	0.12 (0.13)	1.13	0.18 (0.13)	1.20	0.39 (0.14)**	1.48
Emotion Regulation	0.05 (0.12)	1.05	-0.03 (0.14)	0.97 (1.03)	0.06 (0.13)	1.07
ADM	-0.06 (0.10)	0.94 (1.06)	-0.04(0.12)	0.96 (1.04)	-0.05 (0.11)	0.95 (1.05)
DoG	-0.04 (0.10)	0.96 (1.04)	-0.02 (0.12)	0.98 (1.02)	-0.02 (0.11)	0.98 (1.02)
HRV	0.16 (0.10)	1.17	0.00 (0.12)	1.00	-0.02 (0.11)	0.98 (1.02)
Age	-0.11 (0.12)	0.90 (1.11)	-0.09 (0.13)	0.92 (1.09)	-0.20 (0.14)	0.82 (1.22)
SES	-0.07 (0.12)	0.93 (1.08)	0.07 (0.13)	1.07	-0.02 (0.12)	0.98 (1.02)
Gender	1.48 (0.22)***	4.38	-0.45 (0.24)	0.64 (1.56)	0.94 (0.23)***	2.56
	Decreasing		***Moderate Low***		***Moderate Low***	
Flexibility	-0.26 (0.11)*	0.77 (1.30)	-0.14 (0.07)*	0.87 (1.15)	-0.13 (0.09)	0.88 (1.14)
Parent-Rated Inhibition	-0.35 (0.15)*	0.71 (1.42)	-0.22 (0.09)*	0.80 (1.24)	-0.28 (0.10)**	0.76 (1.32)
Stroop Interferences	-0.10 (0.11)	0.91 (1.10)	-0.11 (0.07)	0.90 (1.12)	-0.03 (0.08)	0.97 (1.03)
WMU	0.07 (0.12)	1.07	0.13 (0.07)	1.13	0.09 (0.09)	1.10
Planning	-0.82 (0.12)***	0.44 (2.26)	-0.50 (0.08)***	0.60 (1.65)	-0.61 (0.10)***	0.55 (1.84)
Emotional Reactivity	0.21 (0.14)	1.23	0.14 (0.09)	1.44	0.04 (0.10)	1.04
Emotion Regulation	0.04 (0.12)	1.04	0.02 (0.08)	1.02	-0.03 (0.09)	0.97 (1.03)
ADM	-0.02 (0.10)	0.98 (1.02)	-0.05 (0.06)	0.95 (1.05)	-0.07 (0.08)	0.93 (1.08)
DoG	0.05 (0.10)	1.05	0.07 (0.07)	1.07	0.03 (0.08)	1.03
HRV	-0.08 (0.11)	0.92 (1.09)	0.04 (0.07)	1.04	0.10 (0.08)	1.10
Age	-0.05(0.11)	0.96 (1.05)	-0.24 (0.08)**	0.79 (1.27)	-0.19 (0.09)*	0.83 (1.21)
SES	0.18 (0.11)	1.20	0.08 (0.08)	1.09	0.07 (0.09)	1.07
Gender	1.96 (0.24)***	7.11	-0.13 (0.14)	0.88 (1.14)	0.50 (0.16)**	1.65

Note: *p<.05, ** p<.01, ***p<.001; Total AAB=total aggression and NAAB score; WMU=working-memory updating; ADM=Affective decision-making; DoG=delay of gratification; HRV=heart rate variability; SES=socio-economic status. All predictor variables z-standardized except age and gender.

Table S9

Parameter Estimates of Multinomial Logistic Regression Analysis for the Likelihood to belong the Mentioned Trajectory as Compared to the Low Trajectory for Proactive and Reactive Aggression as well as Non-Aggressive Antisocial Behavior

Predictor	**Proactive Aggression**		**Reactive Aggression**		**NAAB**	
	B (SE)	*OR* (reversed *OR*)	*B (SE)*	*OR* (reversed *OR*)	*B (SE)*	*OR* (reversed *OR*)
	High		***High***		***Moderate low***	
Flexibility	-0.07 (0.11)	0.94 (1.07)	-0.29 (0.11)**	0.75 (1.34)	-0.09 (0.11)	0.91 (1.10)
Parent-Rated Inhibition	-0.46 (0.15)**	0.63 (1.58)	-0.34 (0.13)**	0.72 (1.40)	-0.51 (0.12)***	0.60 (1.66)
Stroop Interferences	-0.25 (0.12)*	0.78 (1.28)	-0.08 (0.11)	0.92 (1.08)	0.01 (0.10)	1.01
WMU	0.05 (0.13)	1.05	0.17 (0.12)	1.19	0.09 (0.10)	1.09
Planning	-0.87 (0.15)***	0.42 (2.39)	-1.13 (0.13)***	0.32 (3.10)	-0.22 (0.11)	0.81 (1.24)
Emotional Reactivity	0.16 (0.14)	1.17	0.32 (0.13)*	1.37	0.69 (0.13)***	2.00
Emotion Regulation	-0.04 (0.13)	0.96 (1.04)	-0.09 (0.13)	0.92 (1.09)	0.18 (0.11)	1.20
ADM	0.01 (0.10)	1.01	0.04 (0.09)	1.04	0.08 (0.09)	1.09
DoG	0.06 (0.12)	1.06	-0.04 (0.10)	0.96 (1.04)	0.12 (0.10)	1.13
HRV	0.07 (0.12)	1.07	-0.18 (0.11)	0.83 (1.20)	0.01 (0.10)	1.01
Age	-0.05 (0.13)	0.95 (1.06)	-0.16 (0.12)	0.86 (1.17)	-0.06 (0.11)	0.94 (1.06)
SES	0.12 (0.13)	1.13	-0.03 (0.13)	0.97 (1.03)	-0.22 (0.10)*	0.80 (1.25)
Gender	1.27 (0.26)***	3.56	1.05 (0.21)***	2.86	0.22 (0.20)	1.25
	Moderate High		***Increasing***		***Increasing***	
Flexibility	-1.80 (0.09)*	0.84 (1.20)	-0.03 (0.11)	0.97 (1.03)	0.11 (0.42)	1.11
Parent-Rated Inhibition	-0.24 (0.12)*	0.79 (1.27)	-0.33 (0.14)*	0.72 (1.40)	-0.42 (0.42)	0.66 (1.53)
Stroop Interferences	-0.14 (0.09)	0.87 (1.15)	0.11 (0.10)	1.12	-0.26 (0.55)	0.77 (1.30)
WMU	0.20 (0.09)*	1.22	-0.04 (0.11)	0.96 (1.04)	0.28 (0.36)	1.32
Planning	-0.76 (0.10)***	0.47 (2.15)	-0.33 (0.13)**	0.72 (1.40)	-0.67 (0.32)*	0.51 (1.95)
Emotional Reactivity	-0.04 (0.11)	0.96 (1.04)	0.02 (0.13)	1.02	1.30 (0.41)**	3.67
Emotion Regulation	-0.12 (0.11)	0.88 (1.13)	-0.16 (0.13)	0.86 (1.17)	0.25 (0.38)	1.28
ADM	-0.02 (0.08)	0.98 (1.02)	-0.04 (0.10)	0.96 (1.04)	0.06 (0.21)	1.06
DoG	-0.06 (0.08)	0.94 (1.06)	0.03 (0.10)	1.03	0.25 (0.36)	1.28
HRV	0.07 (0.08)	1.07	-0.00 (0.11)	1.00 (1.00)	0.20 (0.31)	1.22
Age	-0.18 (0.10)	0.83 (1.20)	-0.27 (0.13)*	0.77 (1.31)	-0.59 (0.33)	0.56 (1.80)
SES	0.07 (0.10)	1.08	-0.07 (0.14)	0.94 (1.07)	-0.43 (0.32)	0.65 (1.53)
Gender	0.37 (0.17)*	1.44	0.77 (0.22)***	2.15	-0.19 (0.72)	0.83 (1.21)
	Moderate Low		***Decreasing***		***Decreasing***	
Flexibility	-0.06 (0.10)	0.94 (1.07)	-0.13 (0.09)	0.88 (1.14)	-0.08 (0.24)	0.93 (1.08)
Parent-Rated Inhibition	-0.14 (0.12)	0.87 (1.15)	-0.27 (0.10)**	0.76 (1.31)	-1.32 (0.30)***	0.27 (3.76)
Stroop Interferences	-0.04 (0.09)	0.96 (1.04)	-0.11 (0.09)	0.90 (1.12)	0.07 (0.17)	1.07
WMU	0.11 (0.10)	1.11	0.12 (0.08)	1.13	0.18 (0.21)	1.20
Planning	-0.64 (0.11)***	0.53 (1.89)	-0.70 (0.10)***	0.50 (2.01)	-0.65 (0.29)*	0.52 (1.91)
Emotional Reactivity	0.05 (0.11)	1.05	0.02 (0.10)	1.02	0.75 (0.30)*	2.12
Emotion Regulation	0.03 (0.10)	1.03	-0.18 (0.10)	0.84 (1.19)	0.23 (0.26)	1.25
ADM	-0.00 (0.09)	1.00 (1.00)	0.02 (0.07)	1.02	0.08 (0.20)	1.08
DoG	0.07 (0.10)	1.08	0.11 (0.08)	1.11	0.12 (0.21)	1.13
HRV	0.11 (0.09)	1.12	0.03 (0.08)	1.03	-0.51 (0.33)	0.60 (1.66)
Age	-0.09 (0.10)	0.91 (1.10)	-0.16 (0.09)	0.85 (1.18)	-0.22 (0.23)	0.80 (1.25)
SES	0.10 (0.10)	1.10	0.07 (0.11)	1.08	-0.25 (0.26)	0.78 (1.28)
Gender	0.21 (0.18)	1.23	0.61 (0.16)***	1.84	0.43 (0.51)	1.53

Note: *p<.05, ** p<.01, ***p<.001; NAAB=non-aggressive antisocial behavior; WMU=working-memory updating; ADM=Affective decision-making; DoG=delay of gratification; HRV=heart rate variability; SES=socio-economic status. All predictor variables z-standardized except age and gender.

S 10 Extended Differential Findings in Single SR Competences Between AAB Trajectories

MNLR findings pointed to strong similarities, particularly regarding the total AAB score that indicated strongest predictive effects. However, to compare mean level differences between all trajectories at one time without the constraint of classes being referenced for each other to answer the present research questions regarding differences between all four trajectories in baseline SR sufficiently, we additionally performed a series of ANOVAs (Supplementary Material Table S11 for further details). Regarding *physical aggression,* pooled ANOVAs extended the MNLR results and further pointed to the relevance of experimentally-measured SR competences. Beyond higher *cognitive* parent-rated inhibition, *behavioral* planning, and proportion of girls, children on the low trajectory also showed more *cognitive* flexibility, less *affective* emotional reactivity, and higher SES than children on all other trajectories. Extending MNLR results, they showed lower *cognitive* Stroop interference than children on the increasing and high trajectory. Regarding the other groups, children on the increasing trajectory showed higher inhibition, planning, and less emotional reactivity than children on the high trajectory, but did not differ from children on the decreasing trajectory. Children on the decreasing trajectory showed more parent-rated inhibition, planning, less emotional reactivity, and higher SES than children on the high trajectory.

Regarding *relational aggression,* pooled ANOVAs extended the MNLR results. Beyond higher parent-rated inhibition and planning, children on the low trajectory also showed lower emotional reactivity than children on all other trajectories. Consistently, they showed higher flexibility and higher age than children on the moderate low trajectory. Regarding the other groups, children on the moderate low trajectory showed higher parent-rated inhibition than children on the moderate high and high trajectories. Children on the moderate high and high trajectories did not differ.

Regarding *proactive aggression*, pooled ANOVAs suggested more fine-grained differences. Beyond higher planning, children on the low trajectory also showed higher parent-rated inhibition and were more frequently girls than children on all other trajectories. Extending MNLR results, they showed higher flexibility and lower emotional reactivity than children on the high and moderate high trajectory; in contrast, differences in *cognitive* updating and Stroop interference remained non-significant. Regarding the other groups, children on the moderate low trajectory showed higher parent-rated inhibition and were more frequently girls than children on the high trajectory. Children on the moderate high trajectory showed higher parent-rated inhibition and lower emotional reactivity and were more frequently girls than boys than children on the high trajectory. Children on the moderate low and moderate high trajectories did not differ.

Regarding *reactive aggression,* pooled ANOVAs largely extended MNLR results, particularly regarding experimentally-measured SR competences. Beyond higher parent-rated inhibition, planning, and proportion of girls, children on the low trajectory also showed less emotional reactivity than children on all other trajectories. Extending MNLR results, they showed higher flexibility than children on the decreasing and high trajectories, lower Stroop interference than children on the increasing trajectory, and higher SES than children on the high trajectory; in contrast, age differences remained insignificant. Regarding the other groups, children on the increasing trajectory showed higher flexibility, planning, and lower emotional reactivity than children on the high trajectory. Children in the decreasing trajectory showed higher parent-rated inhibition, flexibility, planning and lower emotional reactivity, were more frequently girls and showed higher SES than children on the high trajectory. Children on the increasing and decreasing trajectories did not differ.

Regarding *antisocial behavior*, pooled ANOVAs extended MNLR results. Beyond less emotional reactivity, children on the low trajectory also showed higher parent-rated inhibition and planning than children on all other trajectories. Extending MNLR results, they

showed higher flexibility and were more often girls than children on the moderate low trajectory and showed higher SES than children on the decreasing and moderate low trajectories. Regarding the other groups, children on the increasing trajectory showed higher parent-rated inhibition than children on the decreasing trajectory. Children on the decreasing trajectory showed lower parent-rated inhibition and lower planning than children on the moderate low trajectory. Children on the increasing and moderate low trajectories did not differ.

Regarding the *total AAB score*, pooled ANOVAs largely extended these results, particularly regarding experimentally-measured SR competences. Children on all trajectories significantly differed in parent-rated inhibition and planning (low > moderate low > moderate high > high), and emotional reactivity (low < moderate low < moderate high < high). Children on the low trajectory were not only more often girls, but also showed higher flexibility than children on all other trajectories. They also showed lower Stroop interference than children on the moderate low and high trajectories, higher *affective* emotion regulation than children on the high trajectory, were older than children on the moderate low trajectory and showed higher SES than children on the moderate high and high trajectory. Regarding the other groups, children on the moderate low trajectory showed higher emotion regulation and higher SES than children on the high trajectory. Children on the moderate high trajectory showed higher SES than children on the high trajectory. Children on the moderate low and moderate high trajectories did not differ.

Teacher-rated planning (η_p^2 = .08-.18) and parent-rated inhibition (η_p^2 = .05-.11) yielded the largest effects for all AAB measures but antisocial behavior (largest effects observed for planning and gender).

Table S11

Means and Differences between Trajectories of Aggressive and Non-Aggressive Antisocial Behavior Measures and a Total AAB Score in Self-Regulation Competences and Demographic Variables

	**Physical Aggression**						**Relational Aggression**					
	Low	***Increasing***	***Decreasing***	***High***			***Low***	***Moderate Low***	***Moderate High***	***High***		
	M (SD)	*M (SD)*	*M (SD)*	*M (SD)*	*F*	η_p^2	*M (SD)*	*M (SD)*	*M (SD)*	*M (SD)*	*F*	η_p^2
**WMU**	6.23 (1.47)	6.03 (1.40)	5.94 (1.63)	5.77 (1.14)	3.67*	.007	6.23 (1.44)	6.08 (1.57)	6.01 (1.38)	5.68 (1.33)	2.76	.005
**Stroop Interfer**	24.43 (8.29)^{ab}	26.76 (8.89)^{a}	26.51 (10.13)	28.24 (12.97)^{b}	7.61***	.015	24.54 (8.56)	26.08 (9.37)	25.93 (9.35)	24.81 (7.19)	3.20*	.006
**P. Inhibition**	3.61 (0.64)^{ac}	3.26 (0.67)^{ab}	3.22 (0.7)^{c}	2.92 (0.64)^{abc}	32.01***	.076	3.62 (0.65)^{ac}	3.39 (0.66)^{abd}	3.14 (0.67)^{cd}	3.07 (0.62)^{ab}	21.86***	.057
**Flexibility**	16.9 (4.59)^{abc}	14.84 (5.23)^{a}	14.67 (4.94)^{c}	13.92 (4.52)^{b}	21.67***	.041	16.85 (4.61)^{a}	15.4 (5.01)^{a}	15.53 (4.8)	15.22 (5.18)	10.37***	.020
**Em. Reactivity**	2.37 (0.63)^{ac}	2.55 (0.70)^{ab}	2.63 (0.74)^{c}	2.96 (0.65)^{bc}	15.62***	.041	2.36 (0.62)^{abc}	2.53 (0.7)^{b}	2.68 (0.72)^{c}	2.83 (0.68)^{a}	12.44***	.033
**Em. Regulation**	2.20 (0.66)	2.20 (0.69)	2.15 (0.68)	1.94 (0.62)	2.11	.006	2.21 (0.66)	2.16 (0.65)	2.16 (0.72)	1.89 (0.63)	2.37	.007
**Planning**	3.90 (0.82)^{ac}	3.19 (0.82)^{ab}	3.13 (0.81)^{c}	2.74 (0.82)^{bc}	84.88***	.153	3.88 (0.85)^{abc}	3.45 (0.89)^{b}	3.27 (0.84)^{c}	3.07 (0.85)^{a}	38.62***	.075
**ADM**	5.36 (10.97)	5.14 (12.48)	5.88 (13.57)	7.54 (13.03)	0.70	.003	5.73 (11.33)	4.78 (11.43)	4.76 (12.11)	7.62 (15.12)	1.18	.002
**DoG**	2.79 (1.20)	2.73 (1.29)	2.86 (1.29)	2.59 (1.44)	0.70	.001	2.79 (1.2)	2.79 (1.25)	2.63 (1.36)	2.83 (1.36)	0.52	.001
**HRV**	58.32 (27.71)	65.34 (29.69)	58.41 (28.65)	57.07 (25.31)	2.68	.007	58.76 (27.96)	59.52 (28.25)	59 (26.19)	60.1 (30.47)	0.07	.001
**Gender**	1.39 (0.49)^{abc}	1.74 (0.44)^{a}	1.83 (0.38)^{c}	1.90 (0.30)^{b}	74.78***	.129	1.48 (0.5)	1.51 (0.5)	1.46 (0.5)	1.54 (0.51)	0.54	.001
**Age**	8.35 (0.93)	8.25 (1.03)	8.30 (0.98)	8.34 (1.03)	0.63	.001	8.4 (0.94)^{a}	8.14 (0.95)^{a}	8.31 (0.93)	8.5 (0.94)	7.52***	.015
**SES**	5.05 (1)^{ab}	4.79 (1.13)^{a}	4.98 (1.05)^{b}	4.45 (0.93)^{b}	6.53***	.017	5.03 (1.02)	4.98 (1.01)	4.84 (1.02)	4.67 (0.9)	1.98	.005

Table S11 continued

	**Proactive Aggression**						**Reactive Aggression**					
	Low	***Moderate Low***	***Moderate High***	***High***			***Low***	***Increasing***	***Decreasing***	***High***		
	M (SD)	*M (SD)*	*M (SD)*	*M (SD)*	*F*	η_p^2	*M (SD)*	*M (SD)*	*M (SD)*	*M (SD)*	*F*	η_p^2
**WMU**	6.22 (1.46)	6.05 (1.45)	6.12 (1.49)	5.94 (1.59)	1.85	.004	6.27 (1.46)^{a}	5.97 (1.38)	6.14 (1.42)	5.92 (1.61)^{a}	4.31**	.009
**Stroop Interfer.**	24.48 (8.44)	26.32 (8.56)	26.19 (10.37)	25.44 (8.62)	3.93*	.008	24.09 (8.21)^{ab}	27.04 (10.36)^{a}	25.43 (8.3)	26.85 (10.11)^{b}	9.06***	.018
**P. Inhibition**	3.61 (0.64)^{abc}	3.42 (0.68)^{b}	3.36 (0.67)^{ac}	3.11 (0.67)^{bc}	21.12***	.052	3.65 (0.63)^{abc}	3.35 (0.65)^{a}	3.4 (0.64)^{cd}	3.19 (0.69)^{bd}	29.15***	.071
**Flexibility**	15.85 (4.63)^{ab}	14.81 (5.05)	14.09 (5.04)^{a}	14.28 (4.62)^{b}	11.59***	.023	16.1 (4.54)^{b}	15.1 (4.59)^{a}	14.72 (4.89)^{b}	13.34 (5.09)^{ab}	22.26***	.043
**Em. Reactivity**	2.38 (0.63)^{a}	2.50 (0.69)	2.48 (0.68)^{ab}	2.72 (0.72)^{ab}	8.70***	.021	2.34 (0.61)^{ab}	2.53 (0.67)^{a}	2.47 (0.71)^{b}	2.71 (0.7)^{ab}	15.35***	.040
**Em. Regulation**	2.21 (0.65)	2.21 (0.7)	2.13 (0.68)	2.1 (0.65)	1.29	.004	2.24 (0.65)	2.08 (0.64)	2.13 (0.66)	2.13 (0.69)	3.45*	.010
**Planning**	3.94 (0.82)^{abc}	3.38 (0.91)^{b}	3.29 (0.84)^{a}	3.11 (0.91)^{c}	66.38***	.123	3.99 (0.8)^{ab}	3.61 (0.82)^{a}	3.44 (0.88)^{b}	2.99 (0.83)^{ab}	91.49***	.165
**ADM**	5.47 (11.1)	5.67 (12.67)	5.02 (11.86)	6.02 (12.28)	0.22	.000	5.2 (11.08)	5.8 (12.78)	5.96 (11.79)	5.6 (11.79)	0.38	.001
**DoG**	2.79 (1.19)	2.87 (1.19)	2.64 (1.33)	2.84 (1.36)	1.26	.003	2.78 (1.2)	2.87 (1.18)	2.83 (1.25)	2.67 (1.35)	0.91	.002
**HRV**	58.08 (27.75)	61.44 (30.04)	60.29 (27.5)	61.26 (29.28)	1.01	.003	58.69 (27.81)	60.77 (28.98)	61.6 (29.4)	56.63 (26.9)	1.37	.003
**Gender**	1.42 (0.49)^{abc}	1.53 (0.5)^{ac}	1.58 (0.5)^{b}	1.79 (0.41)^{bc}	22.96***	.044	1.38 (0.49)^{abc}	1.61 (0.49)^{a}	1.57 (0.5)^{bc}	1.7 (0.46)^{c}	31.64***	.059
**Age**	8.37 (0.94)	8.26 (0.91)	8.23 (0.98)	8.41 (1)	1.81	.004	8.39 (0.92)	8.15 (0.97)	8.26 (0.91)	8.33 (1.05)	3.38*	.007
**SES**	5.05 (1)	4.97 (1)	4.91 (1.04)	4.87 (1.01)	1.57	.004	5.07 (0.98)^{a}	4.96 (1.1)	5.01 (1)^{b}	4.72 (1.03)^{ab}	5.57**	.014
	**Non-Aggressive Antisocial Behavior**						**Total AAB score**					
	Low	***Increasing***	***Decreasing***	***Moderate Low***			***Low***	***Moderate Low***	***Moderate High***	***High***		
	M (SD)	*M (SD)*	*M (SD)*	*M (SD)*	*F*	η_p^2	*M (SD)*	*M (SD)*	*M (SD)*	*M (SD)*	*F*	η_p^2
**WMU**	6.26 (1.45)	6.18 (1.37)	5.8 (1.28)	6.06 (1.58)	2.01	.007	6.3 (1.44)	6.11 (1.51)	6.12 (1.51)	5.68 (1.26)	3.95*	.009
**Stroop Interfer.**	24.53 (8.49)	25.2 (7.9)	28.91 (9.16)	26.42 (10.4)	5.00**	.015	24.09 (8.27)^{ab}	26.21 (9.08)^{b}	26.03 (8.45)	28.7 (13.57)^{a}	8.27***	.019
**P. Inhibition**	3.63 (0.63)^{ac}	3.07 (0.73)^{ab}	2.6 (0.53)^{abc}	3.13 (0.57)^{c}	60.35***	.076	3.68 (0.62)^{a}	3.44 (0.64)^{a}	3.2 (0.63)^{a}	2.8 (0.66)^{a}	47.92***	.110
**Flexibility**	16.74 (4.52)^{a}	15.86 (5.21)	14.63 (4.67)	15.53 (5.43)^{a}	5.70**	.041	16.14 (4.45)^{abc}	14.59 (4.83)^{c}	14.4 (4.96)^{a}	12.85 (4.82)^{b}	16.99***	.038
**Em. Reactivity**	2.32 (0.6)^{abc}	3.13 (0.51)^{b}	3.09 (0.81)^{a}	2.85 (0.67)^{c}	55.27***	.041	2.31 (0.58)^{a}	2.44 (0.69)^{a}	2.7 (0.71)^{a}	3.09 (0.63)^{a}	32.06***	.079
**Em. Regulation**	2.21 (0.65)	1.97 (0.67)	2.05 (0.78)	2.13 (0.69)	1.95	.006	2.22 (0.64)^{a}	2.17 (0.67)^{b}	2.17 (0.73)	1.87 (0.61)^{ab}	3.99**	.011
**Planning**	3.84 (0.87)^{ab}	3.23 (0.71)^{b}	2.89 (0.86)^{a}	3.45 (0.82)^{a}	23.12***	.153	4.02 (0.8)^{a}	3.54 (0.82)^{a}	3.18 (0.8)^{a}	2.66 (0.81)^{a}	88.21***	.179
**ADM**	5.37 (11.2)	4.36 (8.87)	5.71 (11.14)	5.91 (13.01)	0.19	.001	5.75 (11.37)	4.84 (11.97)	5.45 (12.36)	5.79 (11.29)	0.44	.001
**DoG**	2.8 (1.22)	2.77 (1.23)	2.58 (1.22)	2.91 (1.22)	0.77	.002	2.83 (1.19)	2.82 (1.23)	2.75 (1.34)	2.62 (1.35)	0.54	.001
**HRV**	58.66 (27.77)	65.16 (24.43)	50.43 (24.96)	60.8 (30.37)	1.55	.006	58.24 (28.03)^{a}	62.59 (30.3)^{a}	59.49 (24.93)	55.82 (28.76)	1.62	.005
**Gender**	1.46 (0.5)^{a}	1.45 (0.51)	1.69 (0.47)	1.59 (0.49)^{a}	5.60**	.129	1.4 (0.49)^{abc}	1.57 (0.5)^{c}	1.67 (0.47)^{a}	1.73 (0.45)^{b}	21.52***	.048
**Age**	8.35 (0.93)	8.18 (1.04)	8.18 (0.83)	8.3 (0.92)	0.69	.001	8.37 (0.92)^{a}	8.15 (0.9)^{a}	8.24 (0.99)	8.34 (0.92)	4.17**	.010
**SES**	5.1 (0.99)^{ab}	4.67 (0.81)	4.46 (1.26)^{a}	4.78 (1.07)^{b}	8.96***	.017	5.11 (0.98)^{a}	5.06 (0.98)^{b}	4.84 (1.04)^{a}	4.4 (0.91)^{ab}	9.22***	.024

Note: Analyses of variance: *p <.05, ** p<.01, ***p<.001; P. Inhibition=parent-rated inhibition; WMU=working-memory updating; ADM=Affective decision making; DoG=delay of gratification; HRV=heart rate variability; Gender: 1=female 2=male; SES=socio-economic status. Same letters in hyperscripts indicate significant differences between the respective trajectories in post-hoc analyses (Holm-adjusted). η^2 reported as mean across imputations; inferential tests use Rubin's D1 pooling.

S 12 Appendix

Deviations from the pre-registration:

Hypotheses 1 a-c: Other than stated in the pre-registration, after considering more previous findings, we did not include heart rate variability in Hypothesis 1 on the zero-order correlations between variables in the manuscript, but included negative correlations between relational aggression and all self-regulation competences instead of only inhibition. Instead of computing a variable of total aggression from physical and relational aggression, we computed a total AAB score. We corrected an error: Other than stated in the pre-registration, we did not expect negative, but positive relations between affective decision-making and non-aggressive antisocial behavior. Hypothesis 3: We additionally specified that individuals on trajectories reflecting high AAB show less heart rate variability and more affective decision making. The primary analytical approach, including the latent class growth analyses (LCGA) and the subsequent multinomial logistic regressions (MNLR), was conducted in accordance with our pre-registration. However, to compare mean level differences between all classes/trajectories at one time without the constraint of classes being referenced for each other to answer the present research questions regarding differences between all four trajectories in baseline SR sufficiently, we additionally performed a series of ANOVAs. These analyses were not part of the original pre-registration and should be considered post-hoc/exploratory.
